# Characterization of a relaxase belonging to the MOB_T_ family, a widespread family in Firmicutes mediating the transfer of ICEs

**DOI:** 10.1186/s13100-019-0160-9

**Published:** 2019-05-03

**Authors:** Nicolas Soler, Emilie Robert, Isaure Chauvot de Beauchêne, Philippe Monteiro, Virginie Libante, Bernard Maigret, Johan Staub, David W. Ritchie, Gérard Guédon, Sophie Payot, Marie-Dominique Devignes, Nathalie Leblond-Bourget

**Affiliations:** 1 Université de Lorraine, Inra, UMR1128 DynAMic, F-54000 Nancy, France; 2 Université de Lorraine, CNRS, Inria, LORIA, F-54000 Nancy, France

**Keywords:** Integrative and conjugative elements, Relaxase, MOB_T_, Conjugation, Tn*916*, Firmicutes

## Abstract

**Background:**

Conjugative spread of antibiotic resistance and virulence genes in bacteria constitutes an important threat to public health. Beyond the well-known conjugative plasmids, recent genome analyses have shown that integrative and conjugative elements (ICEs) are the most widespread conjugative elements, even if their transfer mechanism has been little studied until now. The initiator of conjugation is the relaxase, a protein catalyzing a site-specific nick on the origin of transfer (*oriT*) of the ICE. Besides canonical relaxases, recent studies revealed non-canonical ones, such as relaxases of the MOB_T_ family that are related to rolling-circle replication proteins of the *Rep_trans* family. MOB_T_ relaxases are encoded by ICEs of the ICE*St3*/ICE*Bs1*/Tn*916* superfamily, a superfamily widespread in Firmicutes, and frequently conferring antibiotic resistance.

**Results:**

Here, we present the first biochemical and structural characterization of a MOB_T_ relaxase: the RelSt3 relaxase encoded by ICE*St3* from *Streptococcus thermophilus*. We identified the *oriT* region of ICE*St3* and demonstrated that RelSt3 is required for its conjugative transfer. The purified RelSt3 protein is a stable dimer that provides a Mn^2+^-dependent single-stranded endonuclease activity. Sequence comparisons of MOB_T_ relaxases led to the identification of MOB_T_ conserved motifs. These motifs, together with the construction of a 3D model of the relaxase domain of RelSt3, allowed us to determine conserved residues of the RelSt3 active site. The involvement of these residues in DNA nicking activity was demonstrated by targeted mutagenesis.

**Conclusions:**

All together, this work argues in favor of MOB_T_ being a full family of non-canonical relaxases. The biochemical and structural characterization of a MOB_T_ member provides new insights on the molecular mechanism of conjugative transfer mediated by ICEs in Gram-positive bacteria. This could be a first step towards conceiving rational strategies to control gene transfer in these bacteria.

**Electronic supplementary material:**

The online version of this article (10.1186/s13100-019-0160-9) contains supplementary material, which is available to authorized users.

## Background

Horizontal gene transfer (HGT) drives the evolution of bacterial genomes and plays an important role in the adaptation of bacteria to different environments or the colonization of novel niches. Conjugative elements are major actors that trigger HGT [1, 2]. Besides conjugative plasmids, many integrative and conjugative elements (ICEs) in the host chromosome encode their own DNA transfer from donor to recipient cells by conjugation [3–5]. Genome analyses indicate that ICEs are widespread in most phyla of bacteria, and that many of them carry adaptive genes such as antibiotic resistance or virulence genes [3, 4, 6, 7], hence contributing to the dissemination of these traits among bacterial populations. Thus, many Firmicutes have recently become resistant to antibiotics through ICE transfer, including various strains of Clostridia, Enterococci, Staphylococci and Streptococci, some of which are severe human or zoonotic pathogens [8–14]. Although Gram-positive ICEs are more abundant than conjugative plasmids, especially in Firmicutes genomes [7], their molecular mechanism is still poorly characterized. A recent analysis of 124 completely sequenced streptococcal genomes identified 3 major superfamilies of ICEs in streptococci, the most widespread of which being the ICE*St3*/ICE*Bs1*/Tn*916* superfamily [15]. As Tn*916* and its relatives have been involved in the spread of various antimicrobial resistance genes, especially resistance to tetracyclines and macrolides [6, 14, 16], the molecular characterization of the conjugation machinery from ICE*St3*/ICE*Bs1*/Tn*916* superfamily is critical.

Prior to transfer, ICEs excise from the host chromosome as a circular form. As in conjugative plasmids, the initiation of conjugative transfer is likely performed by a multi-protein complex called the relaxosome, that recognizes the origin of transfer (*oriT*) on the excised ICE DNA to be transferred [17]. As demonstrated for conjugative plasmids, the main protein of the relaxosome is the relaxase, a trans-esterase enzyme encoded by ICEs. The relaxase introduces a single-stranded nick at the *nic* site of *oriT*. Whereas the 3′-end of the nick is used to initiate rolling-circle replication of the element, the relaxase remains covalently bound to the 5′-end via a catalytic tyrosine residue [18]. A coupling protein is assumed to recruit the relaxase-DNA complex to a type-IV secretion system (T4SS) encoded by the ICE, providing energy to translocate this complex into the recipient cell [19]. The relaxase is then thought to join the transferred ssDNA at the *nic* site, leading to a re-circularization of the ssDNA in the recipient cell [20].

Relaxases, also termed Mob (for mobilization) proteins, belong to different families of proteins. The canonical relaxases harbor a histidine triad with a ‘HxH’ signature that is known to coordinate a metallic cofactor [21]. They include the MOB_F_, MOB_P_, MOB_L_, MOB_Q_, MOB_V_ and MOB_B_ families [7, 18, 22, 23]. This ‘HxH’ motif is also found in rolling-circle replication initiators of numerous plasmids and viruses, usually termed Rep proteins [24]. Several other families of relaxases have been identified which do not harbor the ‘HxH’ signature and which are unrelated to canonical relaxases [23]. The best described are the MOB_H_ family related to HD-hydrolases [23, 25], and the MOB_C_ family of relaxases that has no typical relaxase motif, but surprisingly has been shown to be related to restriction endonucleases [26]. Additionally, recent observations indicate that several other protein families also function as relaxases, especially in Firmicutes. This is the case of the TcpM protein from the pCW3 conjugative plasmid (*Clostridium perfringens*) that is related to tyrosine recombinases [27]. Furthermore, rolling circle replication (RCR) initiators of the *Rep_1* family have been found to act as a relaxase, being responsible for the mobilization of plasmids by ICEs, in addition to their role in plasmid maintenance [28].

The ICE*St3*/ICE*Bs1*/Tn*916* superfamily of ICEs, which is widespread in Firmicutes, also encodes non-canonical relaxases. These relaxases were unclassified by Garcillan-Barcia et al [23], but were grouped in a family called MOB_T_ in 2011 [7]. Interestingly, putative MOB_T_ relaxases have also been detected in a number of putative integrated and mobilizable elements (IMEs) in streptococcal genomes [29, 30]. Up to now, the activity of only two MOB_T_ relaxases has been demonstrated, namely those encoded by Tn*916* (Orf20) and ICE*Bs1* (NicK). The Orf20 protein was reported to involve the Tn*916* integrase for DNA sequence specificity [31]. However, this result needs to be confirmed because the Orf20 version used lacked the N-terminal helix-turn-helix (HTH) DNA-binding domain due to misidentification of the start codon [32]. The NicK relaxase has been shown to be essential for conjugation and the *nic* site within ICE*Bs1 oriT* was identified [33]. Up to now, no MOB_T_ relaxase has been characterized biochemically or structurally.

MOB_T_ relaxases are non-canonical relaxases that harbor a PF02486 domain. This domain is also found within Rolling Circle Replication (RCR) initiator proteins of the *Rep_trans* family which are involved in the maintenance of many small Firmicutes plasmids [34]. A structural and biochemical characterization of the active site of the *Rep_trans* RCR initiator protein encoded by the pSTK1 plasmid (*Geobacillus stearothermophilus*) has been performed recently [34].

From a structural point of view, 3D structures are available for three canonical relaxase families: MOB_F_ [23, 35–37], MOB_Q_ [23, 38], and MOB_V_ [39]. To date, no 3D structure is known for the non-canonical relaxases (MOB_H_, MOB_C_, and MOB_T_).

In this work, we characterized the MOB_T_ relaxase encoded by ICE*St3*, hereafter called RelSt3. RelSt3 appeared to be essential for ICE*St3* conjugation, and we determined experimentally the *oriT* sequence. The RelSt3 protein was shown to be dimeric and to exhibit the expected relaxation activity that is dependent on a metallic cation, preferentially Mn^2+^. We also investigated the phylogenetic relationships between MOB_T_ and *Rep_trans* proteins, and we propose a 3D model of RelSt3 by homology with the RepSTK1 structure. Critical residues conserved within *Rep_trans* proteins were mutagenized in RelSt3, and we demonstrated their involvement in RelSt3 activity. Our results constitute the first biochemical characterization of a MOB_T_ relaxase.

## Results

### RelSt3 is a MOB_T_ relaxase related to *Rep_trans* RCR initiators

**Fig. 1:**
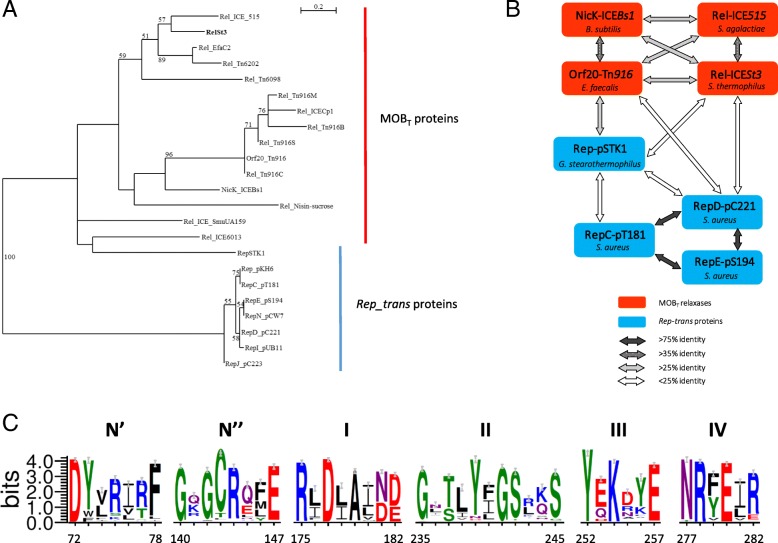
Relationships between *Rep_trans* RCR initiators and MOB_T_ relaxases. **a** Unrooted maximum likelihood tree of MOB_T_ and *Rep_trans* proteins. Fifty-four homologous positions were selected manually for phylogenetic analysis by PhyML. Bootstrap values (100 replicates) above 50% are indicated. The scale bar shows the number of substitutions per site. MOB_T_ proteins: RelSt3 encoded by ICE*St3* from *S. thermophilus* [89], Rel_ICE_515 encoded by ICE*_515_tRNA*^*Lys*^ from *S. agalactiae* [40], Orf20_Tn*916* encoded by ICE Tn*916* from *Enterococcus faecalis* [32], NicK_ICE*Bs1* encoded by ICE*Bs1* from *B. subtilis* [33], Rel_Tn6202 encoded by Tn*6202* from *E. faecalis,* conferring vancomycin resistance [41], Rel_ICE_SmuUA159 encoded by ICE_*SmuUA159_tRNAleu* from *S. mutans*, Rel_Tn6098 encoded by Tn*6098* from *Lactococcus lactis*, and 4 other Tn*916* relaxases from strains of different phyla: Rel_Tn916B from *Bifidobacterium longum*, Rel_Tn916M from *Mycobacterium abscessus*, Rel_Tn916S from *Streptomyces alboniger*, and Rel_Tn916C from *Chlamydia trachomatis*. *Rep_trans* proteins: RepSTK1 corresponds to the *Rep_trans* RCR initiator encoded by the plasmid pSTK1 from *Geobacillus stearothermophilus* [90], whose structure have been solved recently (PDB ID 4CIJ) [34]. Besides the well-characterized RepC (pT181) and RepD (pC221) proteins, *Rep_trans* proteins from 5 other members of the pT181 family of staphylococcal plasmids were included: RepE (pS194), RepI (pUB112), RepJ (pC223), RepN (pCW7) and the Rep from pKH6 plasmid [46]. **b** MOB_T_ relaxases (red boxes) and *Rep_trans* proteins (blue boxes) are linked according to their global sequence identity (see the bottom panel). **c**. Weblogos of the six conserved motifs identified for MOB_T_ relaxase domain. 2016 sequences recovered through CDART analysis with RelSt3 sequence as a query were clustered with a sequence identity of 90%, and this WebLogo was obtained using a representative of each of the 261 clusters obtained. The Chemistry color scheme was chosen for representation of amino-acid residues (WebLogo 3 application): green for polar, purple for neutral, blue for basic, red for acidic, and black for hydrophobic residues. Each motif is numbered on the top, and the motif boundaries in the RelSt3 sequence are indicated on the bottom. See Material and Methods for details

The relationship between MOB_T_ relaxases and *Rep_trans* RCR initiators encoded by small plasmids of the pT181 family has been reported previously [33, 34]. To investigate further this relationship, a selected set of MOB_T_ relaxase sequences including RelSt3 was phylogenetically analyzed together with *Rep_trans* RCR initiator sequences from staphylococcal plasmids (Fig. 1a). The MOB_T_ sequences selected for this analysis include relaxases encoded by the characterized ICEs ICE*Bs1* (NicK), Tn*916* (Orf20), ICE*_515_tRNA*^*Lys*^, ICE*Cp1*, ICE*6013*, Tn*6202*, Tn*6098*, and nisin-sucrose transposon [31, 33, 40–45], and putative relaxases encoded by several Tn*916*-related elements from different bacterial phyla, and by other distantly related ICEs belonging to the ICE*St3*/ICE*Bs1*/Tn*916* superfamily. *Rep_trans* sequences include RepSTK1 whose 3-D structure was recently solved [34], and several members of the Rep proteins belonging to the pT181 family [46]. As expected, RelSt3 groups with the MOB_T_ proteins (Fig. 1a).

The topology of the tree clearly indicated that MOB_T_ and *Rep_trans* from the pT181 family are distantly related (Fig. 1a). Interestingly, the RepSTK1 sequence grouped with MOB_T_ sequences rather than with other *Rep_trans* proteins, even if this topology was not clearly supported by bootstrap analysis. This relationship between RepSTK1 and MOB_T_ proteins was also supported by sequence-based searches for fold similarities using the Phyre2 [47] and iTasser [48] programs. Indeed, the use of any MOB_T_ sequence as query gave the RepSTK1 structure as first hit, with the RepDE structure that belongs to the pT181 family being the second hit [34]. A comparison of sequence identities also illustrates that RepSTK1 is closer to MOB_T_ relaxases than the pT181 cluster (Fig. 1b). As expected, relaxases encoded by Tn*916* and Tn*916*-like elements clustered together among MOB_T_ proteins with a good bootstrap support. RelSt3 is more closely related to the relaxase encoded by ICE*_515_tRNA*^*Lys*^ from *S. agalactiae* than to NicK encoded by ICE*Bs1* from *B. subtilis*. These relationships were also confirmed by multiple alignments of MOB_T_ and *Rep_trans* protein sequences (Additional files 1 and 2: Figure S1 and S2), which allowed the identification in RelSt3 and other MOB_T_ relaxases of the 5 conserved motifs described in *Rep_trans* proteins by Carr et al [34]. Taken together, these data argue in favor of RelSt3 being a bona fide MOB_T_ relaxase.

To identify conserved motifs among MOB_T_ proteins, we recovered 2016 sequences of proteins harboring the same architecture as RelSt3, i.e. with a N-terminal HTH domain and a PF02486 domain (see Materials and Methods for details). To our knowledge, this architecture is only found in proteins encoded by ICEs (and not by plasmids), suggesting that they correspond to relaxases involved in conjugation and not to RCR *Rep_trans* proteins. Multiple sequence alignment followed by WebLogo analysis allowed the identification of 6 conserved motifs (Fig. 1c), distinct from all other motifs found in the other MOB families [22, 23]. Among these motifs, 5 have already been identified in *Rep_trans* proteins, whereas an additional motif, called N″, was found between motifs N′ and I (Fig. 1c). The 3 acidic residues involved in cation coordination in *Rep_trans* proteins [34] were also conserved in the same 3 motifs in MOB_T_ relaxases: motifs N′ (D72 in RelSt3 sequence), I (D177) and IV (D280). In motif III, the catalytic tyrosine identified in *Rep_trans* proteins [49] is also conserved (Y252 in RelSt3 sequence).

### The RelSt3 gene (*orfJ*) is required for ICE*St3* conjugation

If it encodes a functional relaxase, the *orfJ* gene is expected to be essential for ICE*St3* conjugative transfer, as demonstrated for the related gene *nicK* from ICE*Bs1* [33]. We constructed a *S. thermophilus* strain carrying a derivative of ICE*St3cat* deleted for the *orfJ* gene (referred as LMG18311 ICE*St3*Δ*orfJcat*, see Additional file 4, Supplementary Materials and methods). The ICE*St3* conjugation/recombination modules are composed of 17 genes transcriptionally coupled, including *orfJ* at the fifth position downstream of the P_cr_ promoter [50]. Our strategy was to delete the *orfJ* CDS while keeping the predicted *oriT* region and other ICE*St3cat* genes intact, without introducing any foreign sequence. The mating frequency for the LMG18311 ICE*St3cat* [51] as donor strain was 1.0 × 10^− 4^ ± 2.0 × 10^− 5^ transconjugants per donor cell. No transconjugant was observed when three independent clones of LMG18311 ICE*St3*Δ*orfJcat* were used (with a detection level ≥ 10^− 9^ transconjugants per donor cell), thus demonstrating that the *orfJ* gene is required for ICE*St3* conjugation.

### Determination of ICE*St3 oriT*

In order to determine the location of ICE*St3 oriT*, we constructed the pOri1180-*oriT* plasmid harboring the circa 200 bp sequence of the intergenic *orfK/orfJ* region from ICE*St3*, where the conserved *nic* site was previously predicted [52]. This plasmid carrying a spectinomycin resistance marker was tested for mobilization *in trans* by ICE*St3*, and was successfully transferred to recipient cells with a mobilization frequency of 2.5 × 10^− 3^. In contrast, no transconjugant was obtained with the plasmid lacking the *orfK-orfJ* intergenic region (below the 10^− 6^ detection level) (Table 1). These results confirmed that the *oriT* sequence is located between *orfK* and *orfJ* genes in ICE*St3* sequence, and indicated that RelSt3 is able to mobilize a replicon *in trans*.

**Table 1:** ICE*St3* mobilization *in trans* of an *oriT*-harboring plasmid

Donor strain	Recipient strain	Plasmid	ICE*St3* transconjugants frequency	Plasmid mobilization frequency
LMG18311 (ICE*St3cat*)	LMG18311 (pMG36e)	pOri1180	8,8 × 10^−5^	< 10^− 6^
LMG18311 (ICE*St3cat*)	LMG18311 (pMG36e)	pOri1180-*oriT*	2.0 × 10^− 4^	2.5 × 10^−3^

### RelSt3 is dimeric in solution

**Fig. 2:**
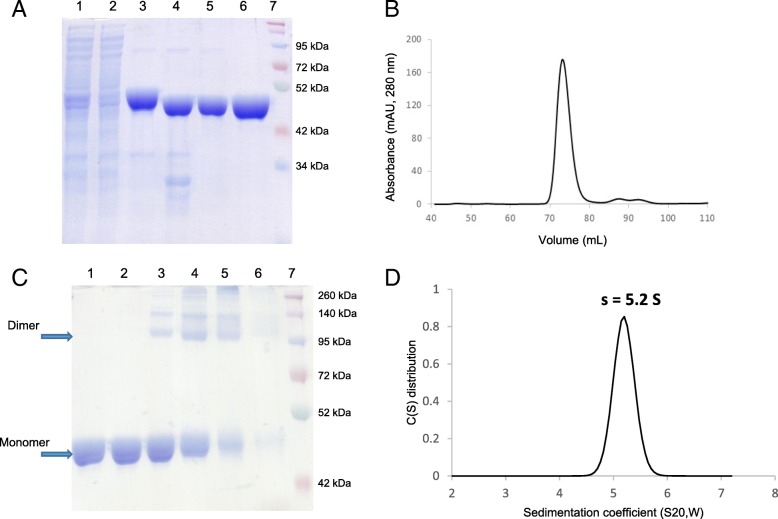
Purification of RelSt3 protein and determination of its oligomeric state. **a** Different steps of RelSt3 purification from *E. coli* (12% SDS-PAGE). Crude extract (lane 1) was submitted to a His-Trap affinity chromatography. Flow-through analysis (lane 2) indicated that most of the protein was retained. The pool of imidazole elution fractions (lane 3) was then digested by the TEV protease to remove His-tag (lane 4), and the sample was then loaded on a second His affinity chromatography. Without tag, the protein was not retained (lane 5), and was concentrated to be injected on a gel filtration column. Finally, the resulting RelSt3-containing fractions were concentrated (lane 6). Lane 7 corresponds to the Spectra Multicolor Broad Range protein ladder (Thermo Scientific). The gel was stained with Coomassie Brilliant Blue. **b** Gel filtration (Sephadex 200) analysis of RelSt3 protein. The elution profile recorded showed a large main peak with a maximum elution volume of 72.4 ml. **c** Glutaraldehyde cross-linking of RelSt3 purified protein. 5 μg of RelSt3 protein were incubated without (lane 1) or with 0.001% (lane 2), 0.01% (lane 3), 0.025% (lane 4), 0.05% (lane 5) or 0.1% (lane 6) of glutaraldehyde. The products were then analyzed by SDS-PAGE (10%), with a Coomassie Brilliant Blue staining. See *Materials and Methods* for details. Lane 7: Spectra Multicolor Broad Range protein ladder. **d** Analysis of the dispersion state of RelSt3 by analytical ultracentrifugation. Sedimentation velocity of RelSt3 was analyzed at 5 μM. The diagram represents the sedimentation coefficient distribution [c(s)]

RelSt3 was over-expressed and purified from *E. coli* using affinity (nickel) chromatography (Fig. 2a) as described in Additional file 4, Supplementary Materials and Methods. RelSt3 was purified to homogeneity, with a yield of about 1.5 mg per g of cell pellet. As the final gel filtration step was performed with a calibrated Superdex 200 column (Additional file 3, Figure S3), the elution volume of the single elution peak (72.4 mL) allowed us to estimate the apparent molecular weight of the protein in solution to be about 88 kDa (Fig. 2b). As the predicted size of RelSt3 is 48.5 kDa, this finding suggested that RelSt3 is dimeric.

The RelSt3 protein was also submitted to chemical cross-linking experiments. To do so, RelSt3 was incubated with increasing concentrations of glutaraldehyde (0.001 to 0.1%, Fig. 2c), and a well-defined band appeared with 0.01% glutaraldehyde around 100 kDa, a molecular mass corresponding to twice the predicted mass of the monomer. At higher concentration of cross-linker, higher molecular mass species appeared.

We next studied the protein homogeneity by velocity sedimentation in order to determine the molecular weight and radius of RelSt3 in solution (Fig. 2d). Data were collected using 1 and 5 μM of protein, which both gave similar results. Most of the RelSt3 absorbance corresponded to a single peak, indicating the absence of higher aggregates, and demonstrating that the oligomeric form of RelSt3 is homogeneous at these protein concentrations. The Sedfit program was used to analyze the c(s) distribution model. The unique peak was associated with a sedimentation coefficient (S20, w) of 5.2 S, a frictional ratio of 1.5 and a hydrodynamic radius of 4.4 nm. Taken together, these data indicate that the RelSt3 protein is an elongated dimer in solution.

### Secondary structure content and stability of RelSt3

**Fig. 3:**
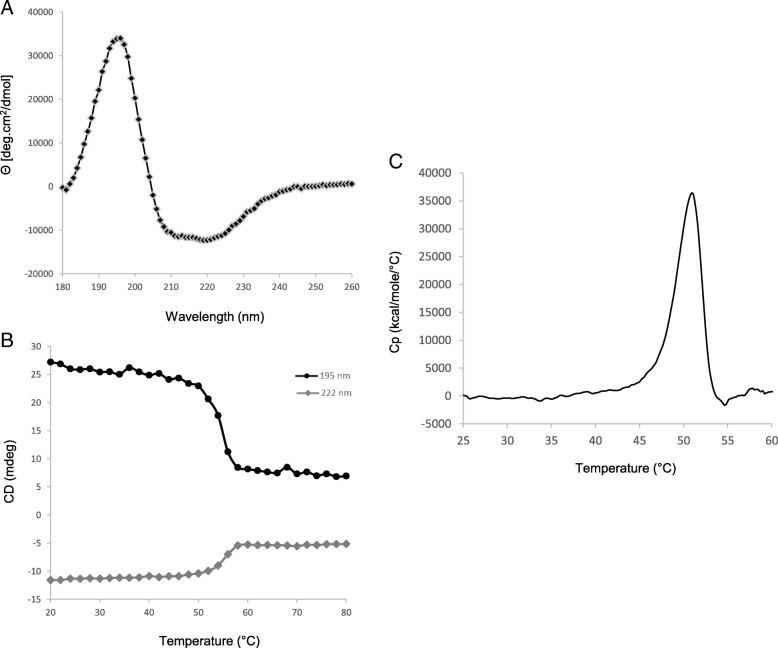
Secondary structure analysis of RelSt3 folding and stability. **a** Far-UV spectrum of RelSt3 protein (12 μM) at 20 °C was recorded as the average of 3 scans from 180 to 260 nm, and converted to mean residue ellipticity. **b** Thermal denaturation curve of RelSt3 protein followed from 20 to 80 °C (1 °C/min) at 195 (black curve) and 222 nm (grey curve). **c** Differential scanning calorimetry analysis was performed with RelSt3

Using various computational methods, the secondary structure of RelSt3 protein was predicted to consist of 41.7% α–helixes, 14.8% β-strands and 43.6% random coils (Table 2). Circular dichroism (CD) was used to analyze RelSt3 folding experimentally. Far-UV spectra were recorded at 20 °C. The spectral data were converted into mean residue ellipticity (Fig. 3a) and showed a maximum at 195 nm, a characteristic feature of β-strand structures, and two minima at 208 and 222 nm, typical of α–helix content [53]. Deconvolution using different methods provided an average content of 39.8% α–helix, 19.4% β-strands, 17.0% turns, and 24.4% unordered (Table 2), indicating that this experimentally determined secondary structure content is in agreement with the in silico prediction. This mixed α/β proportion is similar to those of other CD-characterized relaxase proteins, including MobM from pMV158 and TraA from pIP501 [54, 55], as well as known structures of other relaxases [35, 36, 56, 57].

**Table 2:** Secondary structure content of RelSt3

Prediction programs	α-helix	β-strand	Turns	Unordered
PSI-pred	43.6	10.7	ND	45.7
SABLE	47.3	12.7	ND	40
Jpred4	42.2	14.4	ND	43.4
PredictProtein	38.5	15.1	ND	46.4
APSSP2	36.3	18.8	ND	44.9
Phyre2	42	17	ND	41
Average	41.7 ± 3.9	14.8 ± 2.9		43.6 ± 2.6
Deconvolution methods	α-helix	β-strand	Turns	Unordered
Selcon3	39.7	21.4	17.7	21.8
ContinLL	35.3	26.7	18.4	19.6
CDSSTR	41.0	19.0	18.0	21.0
CDNN	43.2	10.4	14.0	35.3
Average	39.8 ± 3.3	19.4 ± 6.8	17.0 ± 2.0	24.4 ± 7.3

Data and standard deviation are given in percentages. *ND* not determined

To estimate the stability of RelSt3 folding, thermal denaturation was monitored from 20 °C to 80 °C at 222 nm (typical minimum ellipticity of α–helix) and at 195 nm (typical maximum ellipticity of β-strands). Approximately 75% of the ellipticity at 195 nm and 55% of the ellipticity at 222 nm were lost between 20 and 80 °C, with a one-step cooperative transition midpoint at 55 °C (Fig. 3b). After cooling back to 20 °C, almost no ellipticity was recovered (data not shown), indicating that the thermal denaturation is irreversible in our conditions. In conclusion, RelSt3 is a well-folded protein, but is sensitive to denaturation, that is consistent with the observation of protein aggregates during RelSt3 purification trials in presence of ionic force lower than 500 mM NaCl.

Using DSC experiments, we next investigated the overall stability of RelSt3. RelSt3 denaturation occurred in a single step with an enthalpy variation of 144 kcal/mol and a determined Tm of 50.95 °C (Fig. 3c), which is in good accordance with our CD results.

### RelSt3 structure model

**Fig. 4:**
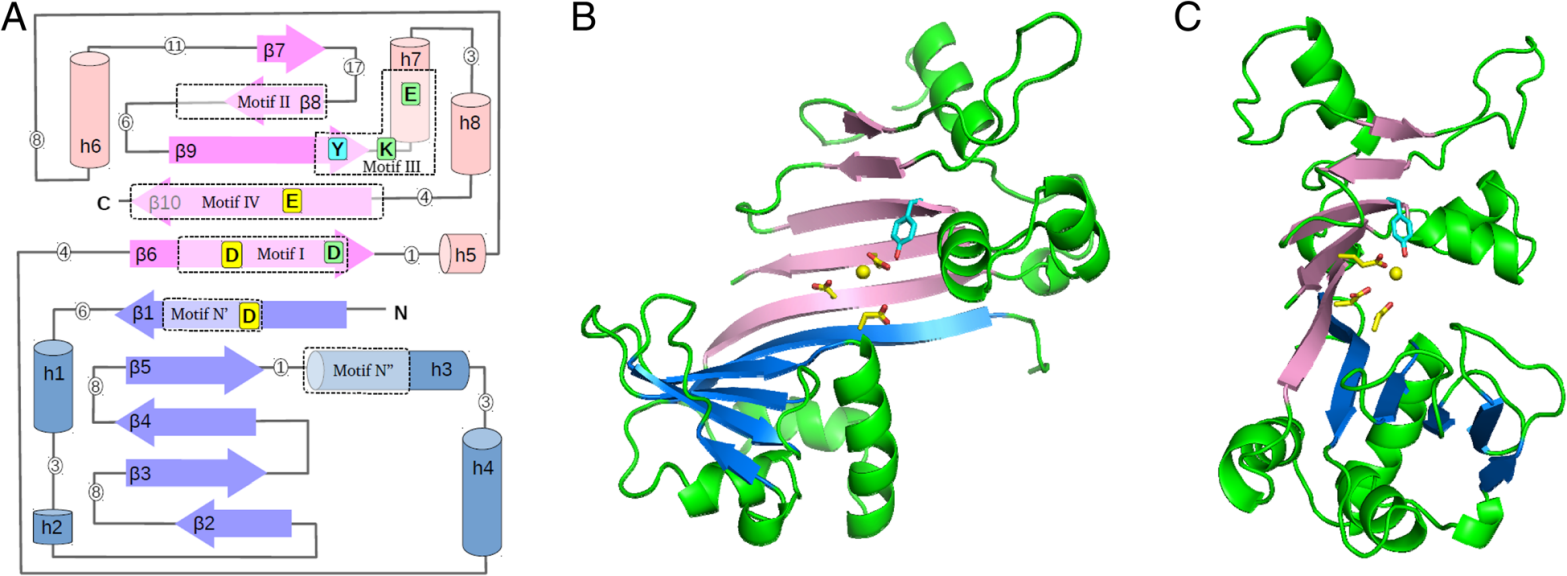
Homology 3-D model of RelSt3 core-domain. **a** Schematic view of the secondary structure of RelSt3 core domain (PF02486). Beta-strands are represented as arrows and helices as cylinders, with lengths proportional to the numbers of residues. The N- and C-lobes, each including a 5-strand beta-sheet, are coloured in blue and pink, respectively. Loops are represented as grey lines, with white circles indicating their length (number of residues). The five motifs conserved both in MOB_T_ and *Rep_trans* proteins are boxed in dotted rectangles. In addition, the motif N″ conserved in MOB_T_ proteins identified in this work is also boxed. The residues mutated in this work are indicated and coloured as follows: yellow for residues chelating the divalent cation, blue for the catalytic conserved tyrosine, green for other conserved residues. **b**. Front view of the homology 3-D model of RelSt3 core-domain. Beta-strands are coloured as in Fig. 4a. Helices and loops are in green. Important residues of the active site are represented as sticks and coloured as in Fig. 4a. **c** side view of the 3-D RelSt3 model, illustrating the crescent-shaped structure with the active site in the centre. The yellow ball indicates the probable location of the divalent cofactor, according to the model

To obtain further structural data of a MOB_T_ relaxase, we built a 3D model of the tertiary structure of the RelSt3 relaxase domain (PF02486) by homology. As phylogenetic analysis indicated that RepSTK1 was the closest protein to RelSt3 for which an experimental structure has been solved (Fig. 1), the X-ray structure of RepSTK1 (PDB 4CIJ) was used as a template. Like the RepSTK1 structure, the RelSt3 model is crescent-shaped, with two lobes of similar secondary structure patterns, each involving 5 beta-strands: β-α-(α)-β-β-β-β-α-α (N-lobe) and β-(α)-α-β-β-β-α-α-β (C-lobe) (Fig. 4) [34]. The underlined elements are absent from the template structure RepSTK1. The two 5-strand beta-sheets are adjacent and form a continuous 10-strand sheet, whose structure and position in the sequence are highly conserved with *Rep_trans* proteins (despite RelSt3 and *Rep_trans* proteins being poorly conserved in sequence, Fig. 1b). In our homology model of RelSt3 relaxase domain, the secondary and tertiary structures are very well conserved compared to the template, and stable over the dynamic simulation. The main differences are a shorter β1-α1 loop (20 vs 27 residues), a longer β9-β10 linker (23 vs 12 residues) including an additional helix (α8), and shorter β7 and β8 strands (3–4 vs 8–9 residues). The parts of our model that correspond to the active site in the template are located at the junction of the two 5-strands sheets, involving the adjacent strands β1 (N-lobe) and β6-β9-β10 (C-lobe), and a short helix downstream of β9 (α7, Figs. 4 and 7).

### In vitro relaxase activity of RelSt3

**Fig. 5:**
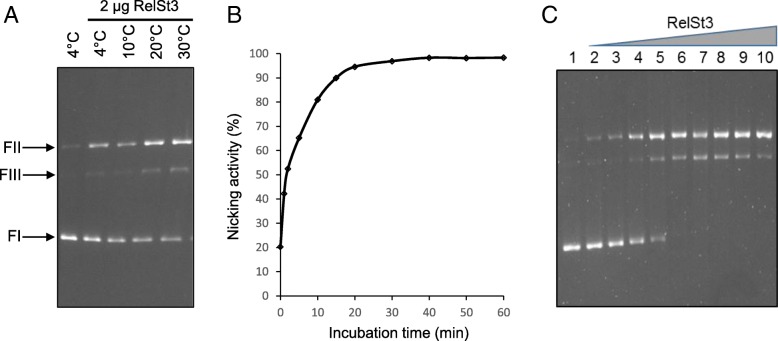
RelSt3 harbours nicking activity. In all assays, 200 ng of pBR322-*oriT* plasmidic DNA were incubated with the indicated amount of RelSt3 protein. **a** RelSt3 nicking activity according to temperature. pBR322-*oriT* plasmid was incubated without or with 2 μg of RelSt3 protein for 20 min at 4, 10, 20, or 30 °C in the presence of 5 mM Mn^2+^. FI: supercoiled plasmid DNA. FII: open circular form. FIII: linear form. **b** Time course in the presence of 2 μg of RelSt3 and 5 mM Mn^2+^ at 30 °C. The nicking activity was established by band quantification monitoring the decrease of FI form. **c** Dose response of RelSt3 nicking activity. DNA was incubated for 20 min at 30 °C with 5 mM Mn^2+^ without (lane 1) or with 0.02 (lane 2), 0.05 (lane 3), 0.2 (lane 4), 0.5 (lane 5), 1 (lane 6), 2 (lane 7), 5 (lane 8), 7 (lane 9) or 10 (lane 10) μg of RelSt3 protein

**Fig. 6:**
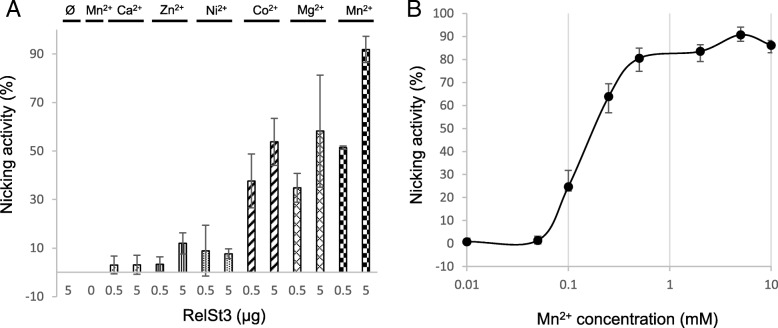
RelSt3 activity in the presence of various divalent cations. **a** RelSt3 relaxation activity in the presence of various divalent cations. 0, 0.5 or 5 μg of RelSt3 (as indicated) were incubated for 20 min at 30 °C with pBR322-*oriT*, without or with 5 mM of the indicated cation (Ca^2+^, Zn^2+^, Ni^2+^, Co^2+^, Mg^2+^ or Mn^2+^). **b** RelSt3 relaxation activity in the presence of various concentration of Mn^2+^. 5 μg of RelSt3 protein were incubated for 20 min at 30 °C with plasmidic DNA with 0.01, 0.05, 0.1, 0.25, 0.5, 2, 5 or 10 mM of Mn^2+^

The relaxation activity of RelSt3 was assayed as previously described by Lorenzo-Diaz et al [54], using a pBR322 plasmid harbouring ICE*St3 oriT* (Additional file 4, Table S2). We were able to demonstrate a nicking activity on this supercoiled substrate (FI), leading to the formation of a relaxed form of the plasmid (FII) (Fig. 5a). To determine the optimal conditions for this relaxation activity, different temperatures (Fig. 5a), incubation times (Fig. 5b), and cations (Fig. 6a) were tested. As shown in Fig. 5a and b, a 20 min incubation at 30 °C of RelSt3 with supercoiled plasmid appeared to give a maximum amount of relaxed form FII. A small amount of linear plasmid was also observed (FIII), probably as a by-product from already nicked DNA. A dose-response experiment was also carried out (Fig. 5c) indicating that the nicking activity is dependent on RelSt3 concentration. As shown for *Rep_trans* proteins [34, 58], no activity was observed without any cationic cofactor (Fig. 6a) indicating that RelSt3 activity depends on the presence of a cationic cofactor. Whereas Ca^2+^, Zn^2+^ and Ni^2+^ were not bona fide cofactors for RelSt3, the presence of Co^2+^, and especially Mg^2+^ or Mn^2+^ is needed for RelSt3 relaxation activity (Fig. 6a). The RelSt3 activity is dependent on Mn^2+^ concentration, and a maximal conversion of form I was observed above 1 mM Mn^2+^ (Fig. 6b).

### Characterization of RelSt3 variants affected in their nicking activity

**Fig. 7:**
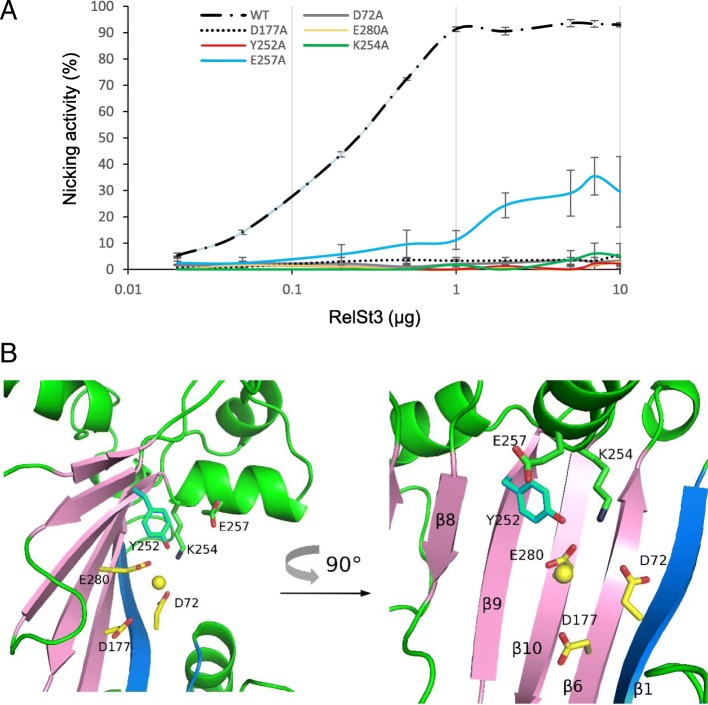
RelSt3 active site characterization. **a** Relaxation activity of RelSt3 point mutants. Dose-response relaxation activity was measured for the point mutants on the catalytic Tyr (Y252) in motif III, and on the adjacent K254 and E257 residues in motif III, and for the point mutants on the 3 conserved acidic residues involved in cation coordination (D72A, D177A and E280A). Relaxation experiments were performed on the pBR322-*oriT* substrate following the standard conditions described in Materials and Methods (Fig. 5c). The standard deviation of 3 replicates is shown. **b** Detailed structure of the homology model of RelSt3 active site. Cartoons are coloured as in Fig. 4. The conserved residues in the active site are represented as sticks and coloured as in Fig. 4. A 90 °C rotated representation is also shown

As mentioned above, multiple alignment of protein sequences (Additional files 1 and 2: Figure S1 and S2) together with our 3D homology model (Fig. 4) identified motifs in RelSt3 similar to conserved motifs proposed for *Rep_trans* proteins [34]. These motifs are also found in other MOB_T_ relaxases (Fig. 1c). Carr et al (2016) demonstrated that 3 acidic residues located in 3 different motifs (N′, I and IV, shown by a star in Additional file 1: Figure S1) are involved in the coordination of the cationic cofactor in *Rep_trans* proteins. As these acidic residues are conserved in the RelSt3 sequence (D72, D177, E280, Fig. 1c and Additional file 1: Figure S1), each one was independently mutated into alanine in order to assess its involvement in RelSt3 activity. In motif III (Additional file 1: Figure S1), the RelSt3 tyrosine Y252 is conserved within *Rep_trans* proteins from plasmids of the pT181 family. The corresponding residue (Y188) of the RepD protein from the staphylococcal plasmid pC221 has already been demonstrated to be catalytic [49]. To confirm that this residue is also catalytic in a MOB_T_ protein, a Y252A variant of RelSt3 was generated. Two other conserved residues were also mutated, K254 and E257, located in the vicinity of the putative catalytic Y252 residue in motif III. All variants were expressed using the same expression vector and with the same purification protocol as for the purification of the WT protein. The nicking activity of each variant was compared to the WT (Fig. 5c and Fig. 7a) with a standard dose-response experiment using Mn^2+^ as cofactor.

As shown in Fig. 7a, each RelSt3 single mutant D72A, D177A and E280A had completely lost the nicking activity, which is consistent with the results obtained for *Rep_trans* proteins modified at these conserved positions [34]. The Y252A variant also showed an absence of nicking activity, suggesting that this residue plays a role of catalytic tyrosine for RelSt3. Concerning the two other conserved residues in motif III, K254 appeared to be essential, whereas E257 was found to be important but not essential for DNA relaxation (Fig. 7a). The positions of these residues in the active site structure model of RelSt3 (Fig. 7b) suggest that E257 could be involved in maintaining the catalytic Tyr in the correct position, whereas K254 could be involved in the stabilization of the acidic residues assumed to coordinate the cation (especially D72 and E280, with Calpha-Calpha distances of 10.5 and 13.0 Å respectively, and maximal side-chain sizes of 6.4 Å, 3.8 Å and 4.8 Å for K254, D72 and E280, respectively).

## Discussion

In this work, we undertook the biochemical characterization of the RelSt3 protein encoded by ICE*St3* of *Streptococcus thermophilus*, a MOB_T_ relaxase. MOB_T_ relaxases are encoded by ICEs belonging to the ICE*St3*/ICE*Bs1*/Tn*916* superfamily found in many Firmicutes [15]. We highlighted here the relationship between the MOB_T_ family of relaxases and the RCR initiator proteins of the *Rep_trans* family, including Rep proteins encoded by the pT181 plasmid family and the structurally characterized RepSTK1 from *Geobacillus stearothermophilus* [34]. Interestingly, an activity of RCR initiation was discovered for several MOB_T_ relaxases encoded by ICEs (Orf20 and NicK). Indeed, these relaxases were shown to be responsible for autonomous RCR initiation starting from the *oriT* [32, 59]. Reciprocally, it has already been demonstrated that RCR initiators involved in the maintenance of small plasmids from Firmicutes can also act as conjugative relaxases. Indeed, these plasmids are still mobilized *in trans* by ICE*Bs1* deleted from its relaxase gene [28]. However, this mobilization was only demonstrated for RCR plasmids encoding a *Rep_1* initiator protein, and was not tested for a RCR plasmid encoding a *Rep_trans* protein.

The purified RelSt3 protein was demonstrated to be homogeneously dimeric. This dimeric state is in agreement with the *Rep_trans* 3D structures solved recently, where the corresponding proteins crystallized as dimers [34]. We demonstrated the relaxation activity of RelSt3 on a plasmidic DNA harboring ICE*St3 oriT*, confirming the functionality of this protein as a relaxase for ICE*St3*. As observed for *Rep_trans* proteins, this single-strand endonuclease activity is dependent on the presence of a divalent metal cofactor that turned out to be preferentially Mg^2+^ or Mn^2+^. Although historically the activity of most relaxases was mainly tested with Mg^2+^ [22, 60–62], when compared with Mn^2+^ the latter was found (i) to be more efficient for nicking activity [54, 63], or (ii) to have a better affinity as measured by micro-calorimetry [54, 64], or (iii) to be the most probable cation according to structural and ICP-MS data [35]. We also noticed that Mn^2+^ was identified in the RepSTK1 structure provided by Carr et al [34]. Accordingly, we observed a slightly higher efficiency in relaxation assays for RelSt3 with Mn^2+^ compared to Mg^2+^.

The secondary structure of the RelSt3 protein deduced from the circular dichroism analysis is very similar to the in silico predictions. A mix of α–helices and β–strands is observed as for most, if not all, characterized RCR Rep and Mob proteins [21, 26, 34, 39]. The thermal stability of a few canonical relaxases has already been studied using circular dichroism, and the addition of a cationic cofactor induced a stabilization of the protein at higher temperatures. Whereas the MobA_R1162 and TraA_pIP501 (MOB_Q_) relaxases present a unique and short transition with a midpoint around 42 °C, indicating a cooperative unfolding [55, 63], the MobM_pMV158 (MOB_V_) relaxase harbors a complex denaturation profile with two transitions (midpoints at 21 and 42 °C) spanning more than 30 °C [54]. In comparison, the MOB_T_ relaxase RelSt3 exhibits a one-step transition as shown by CD and DSC experiments, with midpoints at 55 and 51 °C, respectively. This is clearly higher than observed for other characterized relaxases, indicating that RelSt3 could be intrinsically more stable.

Phenotype analyses of RelSt3 point mutations confirmed the relationship between MOB_T_ and *Rep_trans* proteins. We showed that the catalytic tyrosine of RelSt3 is located in the conserved motif III as in *Rep_trans* proteins. This is the first characterization of catalytic motif for a MOB_T_ relaxase. We also demonstrated the involvement of the 3 conserved acidic residues D72, D177 and E280 (located in motifs N′, I and IV, respectively) in relaxation activity, as demonstrated for RepD variants [34]. These results suggest that both MOB_T_ and *Rep_trans* families (i.e. the PF02486 domain) use 3 distant acidic residues in primary sequence to coordinate their cationic cofactor. In addition, we analyzed the involvement of two other conserved residues of motif III in RelSt3 activity, K254 and E257. Both residues are important for RelSt3 nicking activity, indicating that they could assume a structural role within the active site. Indeed, our 3D structural model of the PF02486 RelSt3 domain suggests that E257 could interact with the catalytic Tyr, and that K254 could interact with the acidic residues D72 and E280 that are assumed to coordinate the cation, maintaining them in the correct position.

The 3D structure model based on RepSTK1 crystal structure revealed a fold that resembles classical HxH endonucleases [21], as it presents a central 10-strand beta sheet (2 × 5 strands) decorated by several alpha-helices. Whereas HxH endonucleases harbour their 3 histidine residues that coordinate the cationic cofactor on two of the central beta-sheets, RelSt3 as well as *Rep_trans* structures instead use 3 acidic residues to do so, each located on a different strand of the central sheet. This correlates with the quite important distance between each of these residues in the primary sequence, lying respectively in motifs N′, I and IV, compared to the classical 3 His that are close to each other in the primary sequence. Another striking difference with HxH endonucleases is that RelSt3 and *Rep_trans* structures harbour their conserved catalytic Tyr residue on one of the central beta-strands (strand beta9 in RelSt3 model), whereas the HxH proteins have their catalytic Tyr residue(s) on an alpha-helix close to the cationic cofactor. These observations suggest that the catalytic assembly orchestrating a Tyr residue and a divalent cation to cleave a DNA substrate arose at least twice in evolution, giving rise to the HxH endonucleases on one hand, and to the *Rep_trans*/MOB_T_ families (harbouring the PF02486 domain) on the other hand, as a remarkable example of evolutionary convergence.

## Conclusions

**Fig. 8:**
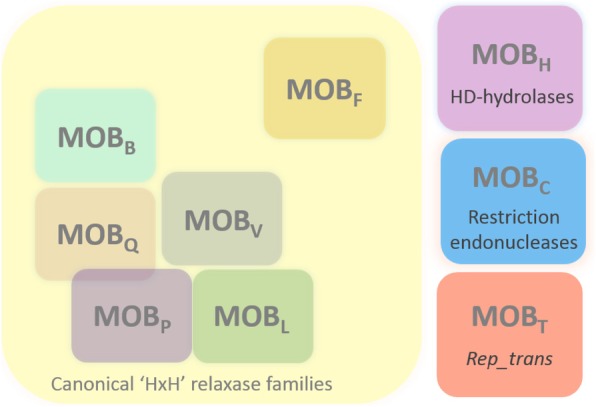
Representation of the main known relaxase families. The canonical families harboring the ‘HxH’ signature are shown on a yellow background. As previously described, the MOB_P_ and MOB_Q_ families partly overlap [23], and the MOB_L_ family is much closer to the MOB_P_ family [22]. MOB_F_ relaxases are distinct from other canonical families since they contain two active tyrosine in their active site. Three other families have been described: the MOB_H_ family, related to HD-hydrolases, the MOB_C_ family related to restriction endonucleases, and the MOB_T_ family related to *Rep_trans* RCR proteins

Even if two MOB_T_ relaxases were identified circa 10 years ago (for Tn*916* and ICE*Bs1* conjugative elements) [31, 33], their affiliation to a unique relaxase family was only pointed out later. This family was unclassified in 2009 [23], proposed in 2011 [7], but surprisingly, it was not mentioned recently [22, 39]. Taken together, our data commit us to propose the MOB_T_ family as a full non-canonical relaxase family alongside the two other previously described non canonical families (MOB_H_ and MOB_C_) (Fig. 8).

In conclusion, we performed the first biochemical characterization of a member of the MOB_T_ family of relaxases, the RelSt3 protein. Its active site characterized here constitutes a potential target for the design of conjugation inhibitors. With this aim in mind, further work is needed, in particular to determine an experimental 3D structure of a MOB_T_ protein.

## Methods

### Phylogenetic analysis and identification of conserved motifs

BLAST searches were performed using the NCBI non redundant (nr) database. The multiple alignment for the phylogenetic analysis was built with the MultAlin interface [65]. Only homologous positions (54 in total) were selected manually for phylogenetic analysis. A tree was constructed with PhyML 3.1 (downloaded from http://www.atgc-montpellier.fr/phyml/versions.php) [66]. The JTT model of amino acid substitution was chosen [67], and a gamma correction with four classes of sites was used. The alpha parameter and the proportion of invariable sites were estimated. The robustness of the tree was tested with PhyML by non-parametric bootstrap analysis (100 replicates).

To identify the conserved motifs in MOB_T_ relaxases, we first constructed a set of MOB_T_ sequences using NCBI’s CDART (Conserved Domain Architecture Retrieval Tool) application, with the RelSt3 sequence as a query [68]. All sequences harboring the RelSt3 architecture (N-terminal HTH domain and PF02486 domain) were aggregated together with the sequences harboring the NicK domain (PF02486 domain only, lacking the HTH domain). A total of 2016 sequences were then clustered using the CD-HIT suite [69], giving rise to 711, 383 and 261 clusters according to sequence identity cut-off of 98, 95 and 90%, respectively. One member of each cluster was selected for sequence alignment with Muscle [70] or ClustalW [71], and the resulting alignments were submitted to the WebLogo 3 interface [72]. The resulting files were very similar for all sequence identity cut-offs (used for CD-HIT clustering) and multiple alignment programs used.

### Bacterial strains and plasmids

The bacterial strains and plasmids used in this study are listed in Additional file 4: Tables S1 and S2. *Streptococcus thermophilus* LMG18311 harboring ICE*St3* labelled with a chloramphenicol resistance gene [51], or its derivative depleted of the *orfJ* gene, were used as donor strains during mating experiments. A strain that derives from *S. thermophilus* LMG18311 and harbors a pMG36e plasmid carrying erythromycin resistance was used as recipient cell. *E. coli* EC101 was used as the host for cloning procedures with pG^+^host9 [73]. For heterologous protein production in *E. coli*, constructs were cloned into the DH5α strain, and over-expression was performed in BL21(*DE3*) cells.

### Construction of ICE*St3ΔorfJcat* and of a mobilizable *oriT*-harboring plasmid

The deletion strategy used to construct the *S. thermophilus* ICE*St3ΔorfJcat* strain is derived from Biswas et al, 1993 [74], with several adaptations described in Additional file 4, Supplementary Materials and Methods. Three independent mutants were verified by sequencing and then used in conjugation assays.

The mobilizable *oriT*-harboring plasmid was constructed using the pOri23 backbone [75], where the Erm^R^ cassette was replaced by a Spec^R^ cassette, giving rise to the pOri1180 plasmid. In order to experimentally identify the *oriT* region of ICE*St3*, we constructed a plasmid encompassing the intergenic region between *orfK* and *orfJ* using *Eco*RI and *Apa*I restriction sites (according to primers described in Additional file 4, Table S3), giving rise to the pOri1180-*oriT* plasmid.

### Mating experiments

Mating experiments were done as described in Dahmane et al, 2017 [76]. Briefly, donor and recipient strains were grown overnight with appropriate antibiotics. Cultures diluted to 1:100 were grown without any antibiotic until mid-exponential phase, and then mixed and concentrated 30 times in LM17 broth. For pOri1180 mobilization experiments, the donor strains were grown in the presence of spectinomycin (500 μg/mL) until mid-exponential phase and washed before the mating experiment. For each mating experiment, three 150 μL aliquots of cells were spread separately on 0.45 μm pore-size nitrocellulose filters (Millipore) deposited on soft agar (0.8%) LM17 plates and then incubated overnight at 42 °C. To select transconjugant cells, bacteria recovered from the filters were directly spread or concentrated 10 times before plating on chloramphenicol and erythromycin, or spectinomycin and erythromycin containing-LM17. After dilutions, the donor and the recipient cells were selected on LM17 plates containing the appropriate antibiotics. After a 24 h-incubation at 42 °C, CFUs counting of three independent biological replicates enabled us to quantify the mating frequency as the number of transconjugants per donor cell.

### Cloning of *orfJ* gene for production in *E. coli* and mutagenesis

Mutagenesis was performed by overlap PCR with the oligonucleotides described in Additional file 4, Table S3. To produce RelSt3 WT or RelSt3 variants, the *orfJ* gene (*wt* or mutated) was cloned in *E. coli* DH5α, using *Nde*I and *Hin*dIII restriction sites into pSKB3 vector (derived from pET28a plasmid, gift from Stephen K. Burley), in frame with a 6 His-tag and a TEV protease cleavage site (N-terminal). Over-expression and purification of recombinant RelSt3 proteins are described in Additional file 4, Supplementary Materials and Methods.

### Protein cross-linking

RelSt3 (5 μg) was incubated in a final reaction volume of 20 μL with various concentrations of glutaraldehyde (0.0, 0.001, 0.01, 0.025, 0.05 and 0.1% *v*/v). The reaction was stopped after 15 min incubation at room temperature by addition of 1 M glycine pH 8.0 to a final concentration of 90 mM. After a 5 min incubation at room temperature, Laemmli loading buffer was added and the samples were heated at 100 °C for 5 min prior to load on a 10% SDS-polyacrylamide gel.

### Analytical ultracentrifugation analysis

Sedimentation velocity assays with RelSt3 were performed at 1 and 5 μM in buffer 20 mM Tris-HCl pH 7.5, 500 mM NaCl. Samples were centrifuged for 16 h at a rotor speed of 42,000 rpm in a Proteomelab XL-I analytical ultracentrifuge (Beckman Coulter) at 25 °C in a four hole rotor AN60-Ti equipped with 12 mm double-sector cells with epon centrepieces. Detection of the protein concentration as a function of radial position and time was performed by absorbance measurements at a wavelength of 280 nm. The following parameters were calculated using Sednterp 1.09 (http://www.jphilo.mailway.com/download.htm) and used for the analysis of the experiment: partial specific volume $\overline{\upsilon}$ = 0.734 mL/g, viscosity η = 0.01054 P, and density ρ = 1.0192 g/mL. Sedimentation velocity data analysis was performed by continuous size distribution analysis c(s) using the program Sedfit 15.1 (http://www.analyticalultracentrifugation.com). All the c(s) distributions were calculated with a fitted frictional ratio *f/f*_*0*_ and a maximum entropy regularization procedure with a confidence level of 0.95.

### Secondary structure prediction, circular dichroism (CD) and differential scanning calorimetry (DSC) assays

Secondary structure content was predicted using the PSI-pred [77], SABLE [78], JPred4 [79], PredictProtein [80], APSSP [81] and Phyre2 [47] programs.

Far-UV CD spectra were obtained from 180 to 260 nm with a Chirascan Plus spectropolarimeter (Applied Photophysics, Ltd., UK) equipped with a Peltier temperature control unit (20 °C). RelSt3 protein was loaded at 20 μM in CD buffer (50 mM sodium phosphate pH 7.0, 500 mM NaF) on a flat quartz cell of 0.1 mm path length. Spectra were recorded with a scan speed of 60 nm.min^− 1^ and 1 nm spectral bandwidth. After an average of 3 scans and subtraction of buffer contribution, raw data were converted to mean residue ellipticity (deg.cm^2^.dmol^− 1^ per residue) using Pro-Data Viewer software (Applied Photophysics, Ltd., UK), and was deconvolved using SELCON3, CONTINLL and CDSSTR algorithms available at the DichroWeb site [82, 83], with the reference data set #3. Data were also deconvolved with the CDNN program [84]. Thermal denaturation curves were followed at 195 and 222 nm from 20 to 80 °C (1 °C/min), and finally a 180–260 nm spectrum was recorded after cooling the sample back to 20 °C.

Differential scanning calorimetry (DSC) experiments were performed with a VP-DSC microcalorimeter (Microcal, Malvern Panalytical, Vénissieux, France). 500 μL of 6.6 μM RelSt3 was incubated with a heating rate of 60 °C/h in buffer A (20 mM Tris-HCl pH 7.5, 500 mM NaCl, 2 mM β-mercapto-ethanol). The experiments were done twice.

### Homology modeling

Ten 3D models of the tertiary structure of the RelSt3 PF02486 domain (residues C61-D285) were built by homology with MODELLER [85], using as a template the X-ray structure of the RepSTK1 protein (PDB 4CIJ, 30% sequence identity with the RelSt3 PF02486 domain). The model with the best Discrete Optimized Protein Energy (DOPE) score [86] was then refined by a molecular dynamics simulation of 100 ns in explicit solvent, and the most representative structure was selected by clustering the conformations selected from each ps of the simulation. Molecular dynamics simulation were performed with the NAMD software [87], using the charmm36 force field [88], and a box of explicit TIP3P water molecules. Counter-ions were added to neutralize the system.

### Supercoiled plasmidic DNA relaxation assays

Relaxation assays were adapted from Lorenzo-Diaz et al, 2011 [54]. Plasmidic DNA (200 ng) of pBR322-*oriT* (Additional file 4, Table S2) was incubated 20 min at 30 °C with increasing amounts of RelSt3 protein (WT or variants, see figure legends), in R buffer [20 mM Tris-HCl pH 7.5, 200 mM NaCl, 5 mM MnCl_2_, 0.05 mM EDTA, 1% glycerol (*w*/*v*) and 1 mM β-mercapto-ethanol] with a final volume of 20 μL. Reactions were stopped by addition of final concentrations of 0.5% SDS and 100 μg/mL of proteinase K, followed by a second incubation at 30 °C for 20 min. Samples were electrophoresed on 1% agarose gel in Tris-Borate-EDTA-1X for 20 h at 2.5 V/cm and the gel was stained with ethidium bromide. Gel pictures were captured using a Bio-Rad XR+ system, and bands were quantified with Image Lab software (Bio-Rad Laboratories). Variations from these standard conditions are indicated in the figure legends.

## Additional files

Additional file 1: **Figure S1.** Alignment of MOB_T_ relaxase representatives with *Rep_trans* RCR initiator representatives. This alignment with reduced number of sequences allows the identification of the different motifs cited in this study. See complete legend in Additional file 4. (RTF 194 kb)

Additional file 2: **Figure S2.** Sequence alignment of MOB_T_ relaxase and *Rep_trans* RCR initiators. This larger alignment with more MOB_T_ and *Rep_trans* initiator sequences illustrates the conservation of the motifs through very distant proteins. See complete legend in Additional file 4. (RTF 1402 kb)

Additional file 3: **Figure S3.** Calibration curve of the gel filtration column. A Sephadex S200 HiLoad 16/60 column (GE Healthcare) was used as a final step of purification of RelSt3 protein. This column was calibrated and allowed us to estimate the apparent molecular weight of RelSt3. See complete legend in Additional file 4. (PPTX 40 kb)

Additional file 4: Supplementary material text. This text includes supplementary Materials and Methods, complete Additional file legends, and tables for bacterial strains, plasmids and oligonucleotides used in this study. (DOC 201 kb)

## Abbreviations

CD: Circular dichroism
DSC: Differential scanning calorimetry
HGT: Horizontal gene transfer
ICE: Integrative and conjugative elements
*oriT*: origin-of-transfer
RCR: Rolling-circle replication
T4SS: Type-IV secretion system
