# Supplementary material for: Characterization of a relaxase belonging to the MOBT family, a widespread family in Firmicutes mediating the transfer of ICEs
Source: Mob DNA. 2019 May 3;10:18. doi: 10.1186/s13100-019-0160-9 (PMC6499999; doi:10.1186/s13100-019-0160-9)
Supplement: Supplementary file 1 — Figure S1. Alignment of MOBT relaxase representatives with Rep_trans RCR initiator representatives. This alignment with reduced number of sequences allows the identification of the different motifs cited in this study. See complete legend in Additional file 4. (RTF 194 kb) [file 13100_2019_160_MOESM1_ESM.rtf]

Figure S1

                                                                                         Motif N'
                                                                                          *
RelSt3        1   MTKISPFQ-IKNFRKQTGLSQKAFAQAVNLPIRTYRSYESGERGLTIDKFRKLKEKLGYYQECHKNNLRAHIDYLRLTFPSLRD-LETFCE------NFL 92  
Rel_ICE_515   1   ---------LKKFRKKTGLKQKEFALSSGLTLKSLRNYEQGKRKLTLEKYQEIKSHFGYLVENDSSRLQVMIDYVRITLKDVRD-LEFFCR------NFL 84  
Orf20_Tn916   1   MNEQTWLQHLKEKRLAYGLSQNRLAVATGITRQYLSDIETGKVKPSEDLQQSLWEALERFNP-DAP-LEMLFDYVRIRFPT-TD-VQQVVE------NIL 90  
NicK_ICEBs1   1   ---------------------------------------MDELKQPPHANRGVVIVKEKNEAVESP-LVSMVDYIRVSFKT-HD-VDRIIE------EVL 52  
RepSTK1       1   ----------------------------------------------------------------MSGLKPCVDWLQVTFKTGQDSVKKCVEKLEKVFEIL 36  
RepDE         1   -------------------------------------------------------MFFTTPQPELS-----FDAMTIVGNLNKTNAKKLSDFMS---TEP 37  
RepC_pT181    1   ----------------------MYKNNHANHSNHLENHDLDNFSKTGYSNSRLDAHTVCISDPKLS-----FDAMTIVGNLNRDNAQALSKFMS---VEP 70  
RepD_pC221    1   -------------------------MSTENHSNYLQNKDLDNFSKTGYSNSRLSGNFFTTPQPELS-----FDAMTIVGNLNKTNAKKLSDFMS---TEP 67  

                                                                             Motif N''                                 Motif I
                                                                                                       *
RelSt3        93  FCHLSEFTDQETRLMNYTHLWQRGNIWIFDFFDKSATNNYQTCLQLSGQGCREMELLLEHKGISWQTFLQNILYAYQD-VRVKRLDIALDELYKGYGHEE 191 
Rel_ICE_515   85  HCAFKEFQPFESKLMNYNHLWKRGDIWIFDFADKHETGNFQITVQLSGRGCRQLELLMETEKFTWHDWLSYLRNSYRDDMNVTRFDIAIDELYLGKDREN 184 
Orf20_Tn916   91  QLKLSYFLHEDYGFYSYSEHYALGDIFVLC---SHELD-KGVLVELKGRGCRQFESYLLAQQRSWYEFFMDVLVAGGV---MKRLDLAIND-------KT 176 
NicK_ICEBs1   53  HLSKDFMTEKQSGFYGYVGTYELDYIKVFY---SAPDDNRGVLIEMSGQGCRQFESFLECRKKTWYDFFQDCMQQGGS---FTRFDLAIDD-------KK 139 
RepSTK1       37  GLNEAEFLPLKNGKYGYKQGVAFQGNPVLAVYYDGADD-MGIHVEMTGQGCRLFEL---HTSINWYELFYRLVYEYEV--NITRLDVAVDD-------FK 123 
RepDE         38  QIRLWDILQTKFKAKALQEKVYIEYDKVKA----DSWDRRNMRVEFNPNKLTHEEML----------WLKQNIIDYMEDDGFTRLDLAFDF-------ED 116 
RepC_pT181    71  QIRLWDILQTKFKAKALQEKVYIEYDKVKA----DSWDRRNMRIEFNPNKLTRDEMI----------WLKQNIISYMEDDGFTRLDLAFDF-------ED 149 
RepD_pC221    68  QIRLWDILQTKFKAKALQEKVYIEYDKVKA----DSWDRRNMRVEFNPNKLTHEEML----------WLKQNIIDYMEDDGFTRLDLAFDF-------ED 146 

                                                                   Motif II       Motif III                      Motif IV
                                                                                 #                           *
RelSt3        192 EHIQIPKLIDKLYAKEIVLDTIRKWNITGGGS--FTDNDNMEANH-GLSLYFGSRQSQLYFNFYEKRYEIARMENISLEESLEIFGIWNRYELRFSDQKA 288 
Rel_ICE_515   185 EQFHLSDMISKYYRHELDFESLRTWNYIGGGSLNFSDMEEIEQNRQGISLYFGSRQSEMYFNFYEKRYEIAKQEGITVEEALEIFELWNRYEIRLSQSKA 284 
Orf20_Tn916   177 GILNIPVLTEKCQQEEC-ISVFRSFKSYRSGELVRKE----EKECMGNTLYIGSLQSEVYFCIYEKDYEQYKKNDIPIEDA----EVKNRFEIRLKNERA 267 
NicK_ICEBs1   140 TYFSIPELLKKAQKGEC-ISRFRKSDFNGSFDL---S----DGITGGTTIYFGSKKSEAYLCFYEKNYEQAEKYNIPLEEL----GDWNRYELRLKNERA 227 
RepSTK1       124 GYFKINTLVKKLKDDEV-TSRFKKARHIENIVIE-------GGETIGHTLYFGAPSSDIQVRFYEKNVQMGMDIDV-----------WNRTEIQLRDDRA 204 
RepDE         117 DLSDYYAMTDKAVKKTIFYGRNGKPE----------------------TKYFGVRDSDRFIRIYNKKQERKDNADVEVMS-----EHLWRVEIELKRDMV 189 
RepC_pT181    150 DLSDYYAMSDKAVKKTIFYGRNGKPE----------------------TKYFGVRDSNRFIRIYNKKQERKDNADAEVMS-----EHLWRVEIELKRDMV 222 
RepD_pC221    147 DLSDYYAMTDKAVKKTIFYGRNGKPE----------------------TKYFGVRDSDRFIRIYNKKQERKDNADVEVMS-----EHLWRVEIELKRDMV 219 
                                                                                 
