# Supplementary material for: Characterization of a relaxase belonging to the MOBT family, a widespread family in Firmicutes mediating the transfer of ICEs
Source: Mob DNA. 2019 May 3;10:18. doi: 10.1186/s13100-019-0160-9 (PMC6499999; doi:10.1186/s13100-019-0160-9)
Supplement: Supplementary file 2 — Figure S2. Sequence alignment of MOBT relaxase and Rep_trans RCR initiators. This larger alignment with more MOBT and Rep_trans initiator sequences illustrates the conservation of the motifs through very distant proteins. See complete legend in Additional file 4. (RTF 1402 kb) [file 13100_2019_160_MOESM2_ESM.rtf]

Figure S2


                                       10        20        30        40        50        60        70        80        90                 
                              ....|....|....|....|....|....|....|....|....|....|....|....|....|....|....|....|....|....|
Rel_ICESt3_Sthermophilus      --------------------------------------------------------MTKISPFQIKNFRKQTGLSQKAFAQAVNLPIRTY 
Rel_ICE515_Sagalactiae        ----------------------------------------------------------------LKKFRKKTGLKQKEFALSSGLTLKSL 
Rel_Tn916_Efaecalis           -------------------------------------------------------MNEQTWLQHLKEKRLAYGLSQNRLAVATGITRQYL 
Rel_ICEBs1_Bsubtilis          ------------------------------------------------------------------------------------------ 
Rel_Tn6009_Kpneumoniae        ------------------------------------------------------------------------------------------ 
Rel_Tn6202_Efaecalis          -----------------------------------------------------------MTNQEIRQLRNRLGLSQQQFADKLHWSKSYL 
Rel_ICESmuUA159_Smutans       ----------------------------------------------------------MLSHADLKRIRLELGFTQRKMAAIIGYSYFNY 
Rel_ICE6013_Saureus           ------------------------------------------------------------------------------------------ 
Rel_Tn6098_Llactis            --------------------------------------------------------------MELAKVRKSFGLSQNDIVKITGLSKSMV 
Rel_Nisin-sucrose transposon  ------------------------------------------------------------------------------MADLLGISLVYY 
Rel_Tn5386                    ---------------------------------------------------------------------------------MAGIRREHL 
Rel_ICECp1                    ---------------------------------------------------------------------------------MAGISREHL 
Rel_EfaC2                     ---------------------------------------MILSHFSTKVVTFFGSRFKAVRGVDLKRYRKELKLKQQELASKLGIERSLI 
Rel_Tn916_Blongum             -----------------------------------------------------MVLNEEQWIKELREKRIAYGISQGRLAVASGITREYL 
Rel_Tn916_Ctrachomatis        --------------------------------------------------MEGFLLNEQTWLQHLKEKRLAYGLSQNRLAVATGITRQYL 
Rel_Tn916_Mabscessus          ------------------------------------------------------MVQRNLDYRLLKDRRNEYGISQNKLATACGLSRPYL 
Rel_Tn916_Salboniger          ------------------------------------------------------MENEKIWIKELKEKRLAYGVSQNKLAVASHITRPYL 
Rel_ICE_SanC238_tRNAleu       -----------------------------------------------------------MTNVELKRIRLELGFTQRKMATMIGYSYYNY 
Rel_ICE_Sga43143_rpsI         ----------------------------------------------------------MISNTDLKRIRLELGFTQRKMATMIGYSYYNY 
Rel_ICE_SintB196_tRNAleu      ----------------------------------------------------------MISNTDLKRIRLELGFTQRKMATMIGYSYYNY 
Rel_ICE_Sdy12394_lysS         --------------------------------------------------------MTIIDGKQLRKFRASLGLKQKEFAEVAGLSLSSL 
Rel_ICE_SparauNCFD2020_rpsI   --------------------------------------------------------MDKLSPLNLKKFRKQTGMTQKQFAESVGISTRTY 
Rel_ICE_SgaUCN34_ftsK         -----------------------------------------------------------MEAYYLQRFRKKTGLNQSDFAKTVGISQSLI 
Rel_ICE_SmiB6_guaA            ------------------------------------------------------MENEKIWIKELKEKRLAYGVSQNKLAVASHITRPYL 
Rel_ICE_SdyRE378_rpsI         --------------------------------------------------------MDKISPFNLKKFRQETGMSQKQFAEAVDLPTRTY 
Rel_ICE_SparasFW213_ebfC      --------------------------------------------------------MDKISPFHIKNFRKQTGLSQKAFAQAVDLPTRTY 
Rel_ICESpn8140                --------------------------------------------------------MDKISPFHIKNFRKQTGLSQKAFAQAVDLPTRTY 
Rel_ICE_Sdy2713_tRNAthr       -----------------------------------------------------------MNPMYLKKFRKKSGLKQKEFAKCIGISQAML 
Rel_dICE_Sag2603_tRNAlys      ----------------------------------------MGHFSEKSGSLILRRSLFIVDAIYLKKFRKKTGLKQKEFALSSGLTLKSL 
Rel_ICE_SgaUCN34_Tn916        --------------------------------------------------MEGFLLNEQTWLQHLKEKRLAYGLSQNRLAVATGITRQYL 
Rel_ICE_Sga2069_rpmG          -----------------------------------------------------------MDVIYLKELRKKMRLNQTNFAKSVGVSRQVI 
RepSTK1-4CIJ                  ------------------------------------------------------------------------------------------ 
RepC_pT181                    ---------------------------------------------------------------------------------MYKNNHANH 
RepD_pC221                    ------------------------------------------------------------------------------------MSTENH 
RepE_pS194                    ----------------------------------------------------------------------------------------MS 
RepI_pUB112                   ---------------------------------------------------------------------------------MSKKEQRIF 
RepJ_pC223                    ---------------------------------------------------------------------------------MSKNNYTNH 
RepN_pCW7                     ---------------------------------------------------------------------------------MSKNNHANH 
Rep_pRS2_Ooeni                ------------------------------------------------------------------------------------------ 
Rep_pK214_Llactis             ------------------------------------------------------------------------------------------ 
Rep_pSP197_Staphpasteuri      ------------------------------------------------------------------------------------------ 
Rep_pLA2_10_Plarvae           ------------------------------------------------------------------------------------------ 
Rep_pBt1-3_Bthuringiensis     ------------------------------------------------------------------------------------------ 
Rep_pBMB2062_Bthuringiensis   ------------------------------------------------------------------------------------------ 
Rep_pMC5_Exiguobacterium      ------------------------------------------------------------------------------------------ 
Rep_pUMNLJ21_2_Ljohnsonii     ------------------------------------------------------------------------------------------ 
Rep_pBC9801_Bcytotoxicus      ------------------------------------------------------------------------------------------ 
Rep_pMC4_Exiguobacterium      ------------------------------------------------------------------------------------------ 
Rep_pRKC30SC2_Lamylovorus     ------------------------------------------------------------------------------------------ 
Rep_pJS-B_Nmeningitidis       ------------------------------------------------------------------------------------------ 
Rep_pAH187_12_Bcereus         -----------------------------------------------------------------------------------------V 
Rep_pFIS3754-01_Fischerella   ------------------------------------------------------------------------------------------ 
Rep_pJTPS1_Ralstoniasol       MQQRDHRQLDLIGDVLHPHGAQLAIVMAAVLRGACCLQSRPAMHRIDDLLKNSRQAELANWYAPRGRTLMRVRPTPSRHLDRTGIAYGVH 
Rep_pNL932024_Llactamica      --------------------------------------------------------------------------------MEEPNEQRVA 
Rep_pFR18_Lmesenteroides      ------------------------------------------------------------------------------------------ 
Rep_pIH01_Lcitreum            ------------------------------------------------------------------------------------------ 
Rep_p9785S_Ljohnsonii         ------------------------------------------------------------------------------------------ 
Rep_pMCCL4_Mcaseolyticus      ---------------------------------------------------------------------------------MNKN----- 
Rep_pQY003_Emundtii           ------------------------------------------------------------------------------------------ 
Rep_p5_Efaecium               ------------------------------------------------------------------------------------------ 
Rep_p41-4_Efaecium            --------------------------------------------------------------------------MKILSRSLFFFEEEQN 
Rep_pJS42_Efaecium            ------------------------------------------------------------------------------------------ 
Rep_pRS1_Ooeni                ------------------------------------------------------------------------------------------ 
Rep_pF03-3_Lpentosus          ------------------------------------------------------------------------------------------ 
Rep_pQJ012_Weissellaconf      ------------------------------------------------------------------------------------------ 


                                      100       110       120       130       140       150       160       170       180        
                              ....|....|....|....|....|....|....|....|....|....|....|....|....|....|....|....|....|....|
Rel_ICESt3_Sthermophilus      RSYESGERGLTIDKFRKLKEKLG--------YYQECHKNNLRAH------IDYLRLTF-------PSLRD------LETFCENFLFCHLS 
Rel_ICE515_Sagalactiae        RNYEQGKRKLTLEKYQEIKSHFG--------YLVENDSSRLQVM------IDYVRITL-------KDVRD------LEFFCRNFLHCAFK 
Rel_Tn916_Efaecalis           SDIETGKVKPSEDLQQSLWEALE--------RF--NPDAPLEML------FDYVRIRF--------PTTD------VQQVVENILQLKLS 
Rel_ICEBs1_Bsubtilis          --MDELKQPPHANRGVVI---VK--------EKNEAVESPLVSM------VDYIRVSF--------KTHD------VDRIIEEVLHLSKD 
Rel_Tn6009_Kpneumoniae        ------------------------------------------ML------FDYVRIRF--------PTTD------VQQVVENILQLKLS 
Rel_Tn6202_Efaecalis          SMIETGKRTINKTAIERINQTFC--------LE--GGILPMQAK------IDFLRIRF--------KIHA------PDQVIEKVLRMNPE 
Rel_ICESmuUA159_Smutans       RNIEQGQRKITKEFEETL---FH--------FLNRKSETKLEGS------VDWLKIRF--------KTLD------FKMVITKVLKLKPA 
Rel_ICE6013_Saureus           -------MTLKNQCTPLTNRGVE-----------RTNKSAVEAV------VDWVQVTF--------HIDP------ISAVIEDVIGLPIT 
Rel_Tn6098_Llactis            SMIDKGERQLNSESEQLL---VD--------YLHRKPKADITAM------IDYLVIRV--------KTLN------YKKFINEVLKIPDY 
Rel_Nisin-sucrose transposon  RKMENGDRPLSKQFEEKIRNSF---------FKKRESSTVFVGT------NDYTNIRF--------QTLN------VREVVSKILGLNVE 
Rel_Tn5386                    SRIEAGRVTLTEDMKHKLLEAVE----------KFNPDNPMFLL------FDYVRIRF--------PTMD------IKHIIKDILKLNIN 
Rel_ICECp1                    NRIEAGKVTLTEDMQDKLMEAVE--------KF--NPDAPMFLL------FDYVRIRF--------PTLD------IQHVIKDILKLNID 
Rel_EfaC2                     SKIESGKRVISKELEQKIINVLN----------LDGGHASVEAK------IDFLRIRF--------KTLD------VRTVIEKLLHMDMN 
Rel_Tn916_Blongum             NKIESGKMKPSKELLETLHKELA--------RF--NPEAPLTML------FDYVKIRF--------PTLD------IQHIIKDILKLNIN 
Rel_Tn916_Ctrachomatis        SDIETGKVKPSEDLQQSLWEALE--------RF--NPDAPLEML------FDYVRIRF--------PTTD------VQQVVENILQLKLS 
Rel_Tn916_Mabscessus          NQIENGGVTASTKTMRKIFNQLE--------SF--NPDLPLSLL------FDYVRIRF--------PTTD------ARKIIQEILHLKFD 
Rel_Tn916_Salboniger          SDIETGKAVPTQQVKEDLLNALE--------RF--NPDNPLEML------FDYVRIRF--------PTND------VSQIIGEVLRLNMD 
Rel_ICE_SanC238_tRNAleu       RNIEQGQRKMTKEFEQTLFH-----------FLNRKSEAKLEST------VDWLKIRF--------KTLD------FKAIITHVLKLRPT 
Rel_ICE_Sga43143_rpsI         RNIEQGQRKMTKEFEQTLFH-----------FLNRKSETKLEST------VDWLKIRF--------KTLD------FKAVITSVLKLKPT 
Rel_ICE_SintB196_tRNAleu      RNIEQGQRKMTKEFEQSLFH-----------FLNRQSETKLEST------VDWLKIRF--------KTLD------FKAVITSVLKLRPA 
Rel_ICE_Sdy12394_lysS         KSYETGRREFTLEKFKEIKTNMG--------YSFSDSPHPLRLM------IDYLRITF-------KNVRQ------LKEFVESYLYVSFN 
Rel_ICE_SparauNCFD2020_rpsI   RSYEDGSRGLSLEKFGQFKAELG--------YHQENAEELIDVH------IDYLRMTF-------MSIRD------LSHFCQTYLHCSFT 
Rel_ICE_SgaUCN34_ftsK         SRYELGKKTLSVETFHKIKSAFG--------YF-DCDKDRLRYM------IDYLRITF-------KSVRD------LEKFTREYLLIPFR 
Rel_ICE_SmiB6_guaA            SDIETGKAVPTQQVKEDLLNALE--------RF--NPDNPLEML------FDYVRIRF--------PTND------VAQIIGEVLRLNMD 
Rel_ICE_SdyRE378_rpsI         RSYETGERGLSIEKFRDLKAKLG--------FHREHEKQSLRAR------IDYLRISF-------PALRD------LESFCENFLLCHLS 
Rel_ICE_SparasFW213_ebfC      RSYETGERGLTIDKFRELKERLG--------YYQECDKNSLRAQ------IDYLRLTF-------PRLKD------LDTFCENFLHCHLS 
Rel_ICESpn8140                RSYETGERGLTIDKFRELKEKLG--------YYQDCDKNSLRAQ------IDYLRLTF-------PRLKD------LDAFCENFLHCHLS 
Rel_ICE_Sdy2713_tRNAthr       SQYESGKKKLSLEKFQEMKTHFG--------YLTDNDSSRLQVT------IDYVRITL-------KSVRD------LEFFCRNFLHCRFK 
Rel_dICE_Sag2603_tRNAlys      RNYEQGKRKLTLEKYQEIKSHFG--------YLVENDSSRLQVM------IDYVRITL-------KDVRD------LEFFCRNFLHCAFK 
Rel_ICE_SgaUCN34_Tn916        SDIETGKVKPSEDLQQSLWEALE--------RF--NPDAPLEML------FDYVRIRF--------PTTD------VQQVVENILQLKLS 
Rel_ICE_Sga2069_rpmG          SQYELGKKPFSLETYRKFKQSFG--------FEE-CESGRLRFM------IDYLRITF-------MSVRD------LEHFTKTFLLIPFK 
RepSTK1-4CIJ                  -------------------------------------MSGLKPC------VDWLQVTFKTGQDSVKKCVE------KLEKVFEILGLNEA 
RepC_pT181                    SNHLENHDLDNFSKTGYSNSRLD--------AHTVCISDPKLS-------FDAMTIVG-------NLNRD------NAQALSKFMSVEPQ 
RepD_pC221                    SNYLQNKDLDNFSKTGYSNSRLS--------GNFFTTPQP-ELS------FDAMTIVG-------NLNKT------NAKKLSDFMSTEPQ 
RepE_pS194                    KKAEEIQAKQSLEKENSNFSKTG--------YSNSRLNRHIMYTPEPKLHFDAMTIVG-------NLNKN------NAHKLSEFMSIAPQ 
RepI_pUB112                   SNQSIGRENSNFVKTGYSNSRLS---------GQTLGKSQPKLS------FDAMTIVG--------NLNKT-----NAKKLSDFMSVDPQ 
RepJ_pC223                    SNHLENHDSDNFSKTGYSNSRLD--------AHFVCTSNPKLS-------FDAMTIVG-------NLNKN------SAKKLSDFMSLDPQ 
RepN_pCW7                     SNHLENHDLDNFSKTGYSNSRLN---------RHTMYTPEPKLS------FDAMTIVG-------NLNKN------NAHKLSEFMSVEPQ 
Rep_pRS2_Ooeni                ------MTKTSIEKPAPSNSRLS---------VKNVYTSSIKWS------IDRLTIVG----ELAPFLPAGYTTNKISGEVKSRITIEDM 
Rep_pK214_Llactis             -----------------------------------MHPQNPKLS------FDAMTIVG-------NLSRD------NAQKLSSFMSIEPQ 
Rep_pSP197_Staphpasteuri      -MISAKVDQFTLTILPSLTNLEG----~~~~~~~~~~~~~~----FAHISSDYSEYVRNRF--------------------EELLQIVPT 
Rep_pLA2_10_Plarvae           ----------MSEIELVPPTTNR--------GVQTPTQNGLIAC------VDWLQVTF--------KIVQ------NTQEIFEIFNLDPT 
Rep_pBt1-3_Bthuringiensis     ----------MEKHIEKL---------------------SLSVC------VDWLEFTF-------VYGEE------FESIC-SFLGLDPT 
Rep_pBMB2062_Bthuringiensis   ----------------------------------------MQVY------LDRLMIKY-------KDVTEKQFSDVLTKISSKQIFLPNT 
Rep_pMC5_Exiguobacterium      --------MKEGNDGGLVRHSVD--------KLSFVADPKDSGA------IDQLE-KY------------------LQEKIMATRQLTKS 
Rep_pUMNLJ21_2_Ljohnsonii     -------------------------------MQKKRIETMLKVQ------IDWLEFTV---------LNT------QLPKVINTLDLGWM 
Rep_pBC9801_Bcytotoxicus      --MFNSKENFQLEIVEPPSSNTG---------VPGTFDIYSIPC------LDWVQVTFKFVQNSQENARR------IISKISSFLGIDEF 
Rep_pMC4_Exiguobacterium      ----------------MENCSID--------KLAIVADPVNADS------VDRLEKYF---------------------QEDLLLGRSMT 
Rep_pRKC30SC2_Lamylovorus     -----------------------------------MENTALTVS------IDWLEFTI---------LNS------QLHEVMKTMELKWD 
Rep_pJS-B_Nmeningitidis       ------------------------------------------------MERDGVL----------------------------------- 
Rep_pAH187_12_Bcereus         FFVSNSKENFQLRTEEPPYSNTG---------VPGCFDIYTIPC------LDWVQVTFKSVQNSQENARK------IVNKISSFFGIDDF 
Rep_pFIS3754-01_Fischerella   MQNAQKSAVVLISKDNFIPEIFQ------------SEDKDPELF------CDWITASG--------RNLS------QNQLSELLNQILIG 
Rep_pJTPS1_Ralstoniasol       GPALARENGMAADRAERSEAGRPTGRSPGPVKPGESPERKAGAI------VDWFKFTFLPDGSISDALEQ------LRKYFHLVFSVPVT 
Rep_pNL932024_Llactamica      VSRKRLRAVIVRLNPRIVTRGLG--------TENRRADRGSSDM-----HCDWYSATI------SDSLHN------ILGYCRNNLGGELR 
Rep_pFR18_Lmesenteroides      ---------MEQEKAQLI---------------------KNHWS------IDMVNVVG-----------N------LRRMPVAFILNNGE 
Rep_pIH01_Lcitreum            ----------------------M--------KQEKTKTVRFRWS------IDMINEVG-----------N------LRRVPVALILQTGE 
Rep_p9785S_Ljohnsonii         MSKEKSEKTRENEEKSVGLSNSR--------PDGQKLLPSVLIS------VDRITVIG-------VPKLN------DDADTSTFMRIIYR 
Rep_pMCCL4_Mcaseolyticus      -------ELEKNWEPALSNSRLG--------VQTTTTP-LPKIN------FDRMTIIG------DLPLDR------VE-HMAEFLGNDPY 
Rep_pQY003_Emundtii           MSKNGTIVNEWEKLTALSNRRLS--------VQTLTTPLKLHWS------IDRITIVG--------KLKENI----YYHTQNDVLILDFE 
Rep_p5_Efaecium               ----------------------------------MDTPLKIQWS------IDRITIVG--------NLKENI----YYHTTHDVLILDFE 
Rep_p41-4_Efaecium            LSKKGTIINEWEKLTAPSNRRLK--------YQSLTLPLKMQWS------IDRITIVG--------KLKENI----YYHTPNDVLILNFE 
Rep_pJS42_Efaecium            -------MNEWEKLNRISNRRLK--------YQSLTLPLKMQWS------IDRITIVG--------KLKENI----YYHTPNDVLILNFE 
Rep_pRS1_Ooeni                ------MNNLDDKKRAPSNSTLT--------TPISGIKKEYRVS------VDRITLTA------DCPLEV------IDGKLSRWMKETHL 
Rep_pF03-3_Lpentosus          --------------MFKLSFTLG--------GKGVFIMRKVTVK------LDRITVSG--------VLPN--------WSLQDIHDETG- 
Rep_pQJ012_Weissellaconf      -------------------------------------MPTNQVS------IDRITVSG--------ELKA------EYLELQRVMNALGS 

                                                                                Motif N'


                                      190       200       210       220       230       240       250       260       270        
                              ....|....|....|....|....|....|....|....|....|....|....|....|....|....|....|....|....|....|
Rel_ICESt3_Sthermophilus      EFTDQ--ETRL---MNYTHLWQRGN---IWIFDFF--DKS---------ATN-------NYQTCLQLSGQG-CREMELLLE--HKGI-SW 
Rel_ICE515_Sagalactiae        EFQPF--ESKL---MNYNHLWKRGD---IWIFDFA--DKH---------ETG-------NFQITVQLSGRG-CRQLELLME--TEKF-TW 
Rel_Tn916_Efaecalis           YFLHE--DYGF---YSYSEHYALGD---IFVLCSH---EL---------DKG----------VLVELKGRG-CRQFESYLL--AQQR-SW 
Rel_ICEBs1_Bsubtilis          FMTEK--QSGF---YGYVGTYELDY---IKVFYSA--PDD---------NRG----------VLIEMSGQG-CRQFESFLE--CRKK-TW 
Rel_Tn6009_Kpneumoniae        YFLHE--DYGF---YSYSEHYALGD---IFVLCSH---EL---------DKG----------VLVELKGRG-CRQFESYLL--AQQR-SW 
Rel_Tn6202_Efaecalis          VFIYK--NYGF---NHYTETYCFSE---IFVFANP--ENL---------DMG----------VMIELRGRG-CREYELVLE--EQQE-TW 
Rel_ICESmuUA159_Smutans       DFFLE--EKSL---YSYRYMVTYGA---IRILYSDSKKKA---------ESG----------TLIDLTGGG-CRELELILI--EQGR-DW 
Rel_ICE6013_Saureus           LFKKR--NSGI---YFYNRGYEFSN---IKLYYSS--DDE---------SMG----------IHLQLTGTG-CREFEHHLQ--QLNK-TW 
Rel_Tn6098_Llactis            YFEQG--QSGG---NGYPFRVEYGE---IKVFYHN--ENI---------DMG----------ARIEFKGGA-CRLFETFLE--EQNR-TW 
Rel_Nisin-sucrose transposon  NFQLN--EYNR---YQYPFYISYGH---INVYYHD--KDI---------KAG----------VLIEMSGQA-CREMEYEFEYHQKQR-TW 
Rel_Tn5386                    YMLHE--DYGH---YKYTEHYYIGD---VVIYTSA--DE----------EKG----------VLLELKGKG-CRQFESYLL--AQER-SW 
Rel_ICECp1                    YMLHE--DYGH---YKYTEHYYLGD---VFVYTSQ--DE----------EKG----------TLLELKGKG-CRQFESYLL--AQER-SW 
Rel_EfaC2                     WFTHE--SRGF---YHYTETFSYSS---IRIFRNP--ENV---------NMG----------IMLDLSGEG-CRQLEEIFE--EDNNRSW 
Rel_Tn916_Blongum             YMLHE--DYGH---YSYTEHYSLGD---IFIYTSA--DE----------EKG----------VLLELKGRG-CRQFESYLL--AQQR-SW 
Rel_Tn916_Ctrachomatis        YFLHE--DYGF---YSYSEHYALGD---IFVLCSH---EL---------DKG----------VLVELKGRG-CRQFESYLL--AQQR-SW 
Rel_Tn916_Mabscessus          YMLHE--DYAF---YSYQEQYVMGD---IVVMLSH--EE----------DKG----------VLLELKGRG-CRQFETFLL--AQKR-SR 
Rel_Tn916_Salboniger          YMLHE--DFGY---YSYPEHYRFGD---IVVLVSH---DV---------SKG----------VLLELKGKG-CRQFENFLL--AQHR-SW 
Rel_ICE_SanC238_tRNAleu       NFFHE--EKSL---YSYSDMVTYGS---IRVLYSHSEKKA---------EAG----------TLIDLTGGG-CREFELLLK--QQGR-NW 
Rel_ICE_Sga43143_rpsI         DFFHE--EKSL---YSYSDMVTYGS---VRVLYSHSEKKA---------EAG----------TLIDLTGGG-CRELELLLK--QQGR-DW 
Rel_ICE_SintB196_tRNAleu      DFFHE--EKSL---YSYSDMVTYGS---IRVLYSHSEKKA---------EAG----------TLIDLTGGG-CRELELLLK--QQGR-DW 
Rel_ICE_Sdy12394_lysS         EFTSQ--ETTM---MTYNHLYKRGD---IWIFDYF--DKE---------ERS-------NYQITLQLSGQG-CRQMELILE--REGI-SW 
Rel_ICE_SparauNCFD2020_rpsI   EFKEF--ETRL---LNYTRLWKRGN---IWLFDFF--DKI---------ETG-------NYQVTLQLSGQG-CREMELVLD--SIDM-IW 
Rel_ICE_SgaUCN34_ftsK         EFGSY--ETKL---MMYTHLWKRGD---IWIFDYH--DKF---------ETN-------NYQITIQLSGSG-CRQMEVMLE--HYGL-TW 
Rel_ICE_SmiB6_guaA            YMLHE--DFGY---YSYPEHYRFGD---IVVLVSH---DV---------SKG----------VLLELKGKG-CRQFENFLL--AQHR-SW 
Rel_ICE_SdyRE378_rpsI         EFSAQ--ETRL---MNYTHLWQRGN---IWIFDFF--DKA---------ETK-------DYQSCLQLSGQG-CRELEVLLE--FKGI-TW 
Rel_ICE_SparasFW213_ebfC      EFTDQ--ETRL---MNYTHLWQRGN---IWIFDFF--DKS---------VTN-------DYQTCLQLSGQG-CRELELLLE--DKGI-TW 
Rel_ICESpn8140                EFTDQ--ETRL---MNYTHLWQRGN---IWIFDFF--DKS---------VTN-------DYQTCLQLSGQG-CRELELLLE--DKGI-TW 
Rel_ICE_Sdy2713_tRNAthr       EFSSV--ESKL---MNYNHIWKRGD---IWIFDYA--DKH---------DTG-------NYQITLQLSGQG-CRQLELLME--QENF-AW 
Rel_dICE_Sag2603_tRNAlys      EFQPF--ESKL---MNYNHLWKRGD---IWIFDFA--DKH---------ETG-------NFQITVQLSGRG-CRQLELLME--TEKF-TW 
Rel_ICE_SgaUCN34_Tn916        YFLHE--DYGF---YSYSEHYALGD---IFVLCSH---EL---------DKG----------VLVELKGRG-CRQFESYLL--AQQR-SW 
Rel_ICE_Sga2069_rpmG          EFHSY--ETKL---MMYNHLWKRGD---IWIFDYH--DKF---------ETN-------NYQITIQLSGSG-CRQLEVMLE--YYDW-TW 
RepSTK1-4CIJ                  EFLPL--KNGK---YGYKQGVAFQGNPVLAVYYDG--AD----------DMG----------IHVEMTGQG-CRLFEL-----HTSI-NW 
RepC_pT181                    IRL-----------WDILQTKFKAKALQEKVYIEY--DKV---------KAD----SWDRRNMRIEFNPNK-LTRDEM----------IW 
RepD_pC221                    IRL-----------WDILQTKFKAKALQEKVYIEY--DKV---------KAD----SWDRRNMRVEFNPNK-LTHEEM----------LW 
RepE_pS194                    IRL-----------WDILQTKFKAKALQEKVYIEY--DKV---------KAD----AWDRRNMRVEFNPNK-LTHEEM----------LW 
RepI_pUB112                   IRL-----------WDILQTKFKAKALQEKVYIEY--DKV---------KAD----TWDRRNMRVEFNPNK-LTSEEM----------LW 
RepJ_pC223                    IRL-----------WDILQTKFKAKALQEKVYIEY--DKV---------KAD----TWDRRNMRVEFNPNK-LTHDEM----------LW 
RepN_pCW7                     IRL-----------WDILQTKFKAKALQEKVYIEY--DKV---------KAD----TWDRRNMRVEFNPNK-LTHEEM----------LW 
Rep_pRS2_Ooeni                FKVFEHFGHCRLSGNGYQMITDDGE---NVAYFERLKFDK---------NKG-----------RLDFNPNKLDSFFEN----------GL 
Rep_pK214_Llactis             IRL-----------WDILQTKFKAKALQEKVYIEY--DKV---------KAD----TWDRRNMRVEFNPNK-LTHEEM----------IW 
Rep_pSP197_Staphpasteuri      YSSAELMNGGV---RNYDTVYSYGF-EKAKIFFKYSSSNV---------SNG----------ISIEFKARA-LREYLKAYRIKKSDSMDV 
Rep_pLA2_10_Plarvae           YFKDF--PTGK---YGYSSHLRMSN---IAIYYGG--RHA---------NMG----------IHVEMTGQG-CRLYES-----LEGSLSW 
Rep_pBt1-3_Bthuringiensis     VFSKE--IDGFHKSYGYLSRYSFEE---IHILMHG--ADN---------RSR------------IIMSGSG-CRWFETLSS-----V-GW 
Rep_pBMB2062_Bthuringiensis   PIRSE--HGTSVRDYHRVIHIGYGE-GAVYIGWKHNSEKE---------KDS--------YDMKVDFNPSK-FENNELQKD-------SY 
Rep_pMC5_Exiguobacterium      HDPYR---------YMYILPMKLGT---VSIADKN--AKL---------GKR----------LRLEFNPNR------------ATSD-DV 
Rep_pUMNLJ21_2_Ljohnsonii     DFSPL--AKRR---FGYHNQLKWQGGSIFVMFTAN--SDVVDLDTKINPQSG----------IHVMITGQG-CRQYS-----------AK 
Rep_pBC9801_Bcytotoxicus      LFKHC--DDGL---HGYRKSYKFLGQNTFQLLFDA--PS----------NMG----------IHLILTGSM-LKLIRSDYK--KDDI-QL 
Rep_pMC4_Exiguobacterium      AFPYR---------YMYKLPFHAGV---VMIADRN--DKV--------------------RQLRFEFNPKH------------SKRD-DV 
Rep_pRKC30SC2_Lamylovorus     DFSKL--SKGK---FGYNNQIKWSDGSIFIMFTAK--DEDVNESTLINPKSG----------VHVMITGKG-CRQYSVNH--------DL 
Rep_pJS-B_Nmeningitidis       -----~~~~~~~~---YGQVYIGGQ------------------------QET----------ILVEMTGKG-CAVAEE----------GW 
Rep_pAH187_12_Bcereus         LFEHF--DEGL---HGYRKSYKFLGQNTFQLLFDA--PS----------NMG----------IHLILTGSM-LKMLRSDYG--TSDI-RL 
Rep_pFIS3754-01_Fischerella   GFWNKNFDCEK---YDSNTTLFRGG----AVYDSTYQNELGAKLKVLTKEEG--------YTVLLTLSGKV-LSQLID-----RNDLLSV 
Rep_pJTPS1_Ralstoniasol       MKPAG---KGF---RRYEFSYD------LLAFINGETMKLGIVACGGEHVGG---------TILVDWPGQV------------FTAIGDW 
Rep_pNL932024_Llactamica      FG-----SRGR---YGYQKSCFLDD------------------------ENG-------NPNVSILWGGNGGANPNASASG--LKAA-DF 
Rep_pFR18_Lmesenteroides      WICRR--ENYM---WQYIRKFENGD---TEVLITVQRLKF---------HKH------FDDPVWVQYNPNH-LENDDLKQL--ERIM--- 
Rep_pIH01_Lcitreum            WICRR--ENYM---WQYVRRYENGD---IETLITVQRLKF---------HKH------FDNPVWVQYNPNH-LRKEDI------------ 
Rep_p9785S_Ljohnsonii         KWKKE----------SFFKLTTSGN------IVNPDTDENIAYFEVPSHEQG---------KIRVDYNPKR-LREEPEA---------EW 
Rep_pMCCL4_Mcaseolyticus      VNL-----------WEKMNNRFKGKALNEKVYIEH--DRL---------KADAWNR----RNFRIEFNPNN-LSDDEK----------LW 
Rep_pQY003_Emundtii           QLMRL--NEG----NGYLKAVGNNG---WQLLDQY--EENIAYIEILKWQEG---------KGRIDFNPSK-INQFLAG---------SM 
Rep_p5_Efaecium               QLMRL--NEG----NGYLRSVSNNG---WQLLDQH--EENIAYIEILKWQEG---------KGRIDFNPNK-ISQFLAS---------SM 
Rep_p41-4_Efaecium            QLMRL--NEG----NGYLRSVSNNG---WQLLDQH--EENIAYIEILKWQEG---------KGRIDFNPNK-ISQFLAS---------SM 
Rep_pJS42_Efaecium            QLMRL--NEG----NGYLKAVGNNG---WQLLDQH--EENIAYVEILKFHEG---------KGRIDFNPNK-INQFLSG---------SM 
Rep_pRS1_Ooeni                FIPLS--SGGF-----KVIEDNLADTPEQVAFVEY--TQF---------QKN---------RIRIDFNPNHSMT---------TKGG-QW 
Rep_pF03-3_Lpentosus          -LIPR---DGA---MFLEREDKDGN-TENMAFMAE--SPF---------QRE---------HWRLDFNPAN-LTADETAVL--GRVI--- 
Rep_pQJ012_Weissellaconf      SWELS---GGV---FRLIREYPNGD-TENIAYYAE--NAF---------QAG---------SWRLDFNPNK-LTDEEKLEM--KRAI--- 

                                                                                                  Motif N''


                                      280       290       300       310       320       330       340       350       360        
                              ....|....|....|....|....|....|....|....|....|....|....|....|....|....|....|....|....|....|
Rel_ICESt3_Sthermophilus      QTFLQNIL-------YAYQDVRV------------KRLDIALDELYKGYGHEEEHIQIPKLIDKLY-AKEIVLDT-IRKWNITGGGSFTD 
Rel_ICE515_Sagalactiae        HDWLSYLR---N---SYRDDMNV------------TRFDIAIDELYLGKDRENEQFHLSDMISKYY-RHELDFES-LRTWNYIGGGSLNF 
Rel_Tn916_Efaecalis           YEFFMD---------VLVAGGVM------------KRLDLAINDK-------TGILNIPVLTEK-C-QQEECISV-FRSFKSYRSGELVR 
Rel_ICEBs1_Bsubtilis          YDFFQD---------CMQQGGSF------------TRFDLAIDDK-------KTYFSIPELLKKA--QKGECISR-FRKSDFNGSFDLS- 
Rel_Tn6009_Kpneumoniae        YEFFMD---------VLVAGGVM------------KRLDLAINDK-------TGILNIPVLTEKC--QQEECISV-FRSFKSYRSGELVR 
Rel_Tn6202_Efaecalis          TEFFWRLY---ET--NLFDNHRM-------IDTKITRIDLALDEQVSLL---YPSYDLFELKAK-Y-EQGLVDTT-FRNFDFTGGIVVKN 
Rel_ICESmuUA159_Smutans       FSFLHDVF-------LFAEQERKDRLLED--FLAFPRFDIALDELYKE----TGNLDLFDIKARVF-DNKIIMKR-TRTFTAIEGLKKVD 
Rel_ICE6013_Saureus           QDFFDK---------CLSVNANF------------TRIDIAIDDY-------KTYLKVPLLIKK-A-EKAECVSR-FRAGSAINGFNLS- 
Rel_Tn6098_Llactis            QDFLKDVI---NYSFEATRINGVDDPIEARKFLKFKRLDIALDERFNE----KGNYNLMALWEKV--RKGQIEMK-LKGFRPEEEFKMSD 
Rel_Nisin-sucrose transposon  YDFFND---------CFLYANKK--APENDDFVKITRFDLALDEQYNPQ---EGNFDLFKLLTSA--REGRWNGR-KQNYSAVLGGRRTK 
Rel_Tn5386                    YDFFMD---------ALVEGGVM------------KRLDLAINDR-------TGLLDIPELIKKC--ENEECISK-FRSFKNYGSGELVK 
Rel_ICECp1                    YDFLMD---------ALVEGGVM------------KRLDLAINDR-------AGILDIPDLTAK-C-NREECVSL-FRSFKSYASGELVK 
Rel_EfaC2                     TEFFRSLYDDDIFGQGILVDTKI------------TRIDIALDELIVKG---QENFDLYVLKEKM--EQGLVDTT-FKNFDFSGGFVYEN 
Rel_Tn916_Blongum             YDFLMD---------ALVDGGVM------------KRIDLAINDH-------TGILDIPELAEK-C-RKREYIGK-SRSYKFYQSGELIK 
Rel_Tn916_Ctrachomatis        YEFFMD---------VLVAGGVM------------KRLDLAINDK-------TGILNIPVLTEK-C-QQEECISV-FRSFKSYRSGELVR 
Rel_Tn916_Mabscessus          YDFFED---------CLKAGGVM------------KRLDLAINDL-------VGLLDIPDLTKK-C-QKEECISL-FRTFKSYRSGELLK 
Rel_Tn916_Salboniger          YGFFQD---------CMEHKGIF------------KRLDLAINDK-------TGILNIPELAKK-C-KQEECISV-FRSFKNYRSGELVH 
Rel_ICE_SanC238_tRNAleu       FSFLHDVF-------LFAEQERKDRPL--EDFLAFPRFDIALDELYKK----SGNLDLFDIKNRIF-DNKIIMKK-LKTFTAIEGLKKVE 
Rel_ICE_Sga43143_rpsI         FSFLHDVF-------LFAEQERKDRPL--EDFLAFPRFDIALDELYKK----SGNLDLFDIKNRIF-DNKIIMRK-LRTFTAIEGLKKVE 
Rel_ICE_SintB196_tRNAleu      FSFLHDIF-------LFAEQERKDRPL--EDFLAFPRFDIALDELYKK----SGNLDLFDIKNRIF-DNKIIMRK-LRTFTAIEGLKKVE 
Rel_ICE_Sdy12394_lysS         RDFLEKML-------YERSDMKV------------TRIDLALDELYRGYDQEETHFHLSDMINKVY-QHLVTFDR-LRTWSHIGGGNLNS 
Rel_ICE_SparauNCFD2020_rpsI   QEFLQALL-------FDLTDSRV------------TRLDIAMDERYLGHDREDEQFLLSDMIAKVY-KDEVSFKN-IRTWNHIGGGNLRN 
Rel_ICE_SgaUCN34_ftsK         RDLLANMR---S---AFGDSMKV------------TRLDIAIDELYLGVGRENEQFQLEDLISKYY-KKELYFDK-LRKWNYIGGGSLGS 
Rel_ICE_SmiB6_guaA            YDFFQD---------CMEHKGIF------------KRLDLAINDK-------TGILNIPDLAKK-C-KQEECISV-FRSFKNYRSGELVH 
Rel_ICE_SdyRE378_rpsI         QTFLQNLF-------YAYEDCRI------------KRLDIALDELYKGFGKEDQQIHIPKLIEKLY-AKEIVLNT-FRKWNVTGGGSFID 
Rel_ICE_SparasFW213_ebfC      QIFLQNIL-------YSYEDVRV------------KRLDIALDELYKGYGHEDEQIQIPKLIDKLY-SKEIVLDT-IKKWNITGGGSFTD 
Rel_ICESpn8140                QIFLQNIL-------YSYEDVRV------------KRLDIALDELYKGYGHEDEQIQIPKLIDKLY-SKEIVLDT-IKKWNITGGGSFTD 
Rel_ICE_Sdy2713_tRNAthr       LDWLIYLR---R---SYRDEMNV------------TRFDLAIDELYLGKDRENEHFLLSDMIAKYY-HHELAFES-LRTWNYIGGGALNF 
Rel_dICE_Sag2603_tRNAlys      HDWLSYLR---N---SYRDDMNV------------TRFDIAIDELYLGKDRENEQFHLSDMISKYY-RHELDFES-LRTWNYIGGGSLNF 
Rel_ICE_SgaUCN34_Tn916        YEFFMD---------VLVAGGVM------------KRLDLAINDK-------TGILNIPVLTEK-C-QQEECISV-FRSFKSYRSGELVR 
Rel_ICE_Sga2069_rpmG          QDLLANMR---L---AYGADMKV------------TRLDIAIDEVYQGVGQEKEQFQLQDMISKYY-KQELYFDK-MKKFNFIGGGSLIY 
RepSTK1-4CIJ                  YELFYRL--------VYEYEVNI------------TRLDVAVDDF-------KGYFKINTLVKK-L-KDDEVTSR-FKKARHIENIVIE- 
RepC_pT181                    LK--QNII-------SYMEDDGF------------TRLDLA--------------FDFEDDLSDYY-AMSDKAVK-KTIFYGRNG----- 
RepD_pC221                    LK--QNII-------DYMEDDGF------------TRLDLA--------------FDFEDDLSDYY-AMTDKAVK-KTIFYGRNG----- 
RepE_pS194                    LK--QNII-------DYMEDDGF------------TRLDLA--------------FDFEDDLSDYY-AMTDKSVK-KTIFYGRNG----- 
RepI_pUB112                   LK--QNII-------DYMEDDGF------------TRLDLA--------------FDFEDDLSDYY-AMTDKAVK-KTVFYGRNG----- 
RepJ_pC223                    LK--HNII-------DYMEDDGF------------TRLDLA--------------FDFEDDLSDYY-ALSEKALK-RTVFFGTTG----- 
RepN_pCW7                     LK--QNII-------DYMEDDGF------------TRLDLAFDFE----------YDLSDYY-----AMTDKSVK-KTIFYGRNG----- 
Rep_pRS2_Ooeni                KQFIS----------DLFITPHF------------SRADIAADI-----------FDVPDEKVVSY-RLGEPVGN-TFFYGKSGD----- 
Rep_pK214_Llactis             LK--QNII-------DYMEDDGF------------TRLDLA--------------FDFEDDLSDYY-AMTDKAVK-KTIFYGRNG----- 
Rep_pSP197_Staphpasteuri      HRILSLL--------ADEFNIRL------------SRLDIAIDFV-------DEDVLVTKIYDDLL-SEELIIKN--KGNRIISDDKIRT 
Rep_pLA2_10_Plarvae           DKLLYKI--------IYGYDVNI------------TRFDLAIDDV--RYNEDKPYFTVKKLKRK-L-KDGECVSK-FKKAREMNTLDIA- 
Rep_pBt1-3_Bthuringiensis     YGLFDRI--------AFADEHGF---S----WIKVDRLDIAIDDF-------VGYFSVKKLKSKI--KRRECLSR-WKTSFVVETFDLE- 
Rep_pBMB2062_Bthuringiensis   EKVFETVF---HTLNAVLKSNKR----------VVYGMDIAFDIE----------RHMSDIVSY-S-KTGKQQDR-HKG----------- 
Rep_pMC5_Exiguobacterium      RQMYINII-------ATMKYPQL------------TRVDLA--------------FDYAENLS----DIRWVDKK-GRPSSLYRNGKYV- 
Rep_pUMNLJ21_2_Ljohnsonii     HDLMHLIR---VL--SAHERVNF------------TRIDLAVDDF-------ESKIVSYDKIHDAA-IKGHFTSR-WSKWDEVTSRQTS- 
Rep_pBC9801_Bcytotoxicus      LKFLT----------SVSKGFHF------------SRIDVAKDDV-------SGSVSIKKIARY-I-KDGNLTTR-FRGGHQIKKFKLVG 
Rep_pMC4_Exiguobacterium      EAMYKRIL-------SFMKYPEI------------KRLDLA--------------FDYAENLS----EMRWLDSK-SRPFNVHH------ 
Rep_pRKC30SC2_Lamylovorus     ISLIKRL--------YSLPRVNF------------SRIDLAIDDF-------ESKIINYDRIHEAA-INGHFTSR-WSKWDEINSRQTS- 
Rep_pJS-B_Nmeningitidis       EERLYRFL---TA--DTTYNAKI------------TRCDVAKDFY--------ENEISPDTAWEMY-EQGKFDKRGKRPLVGKLGDDWL- 
Rep_pAH187_12_Bcereus         LKFLS----------SISKEFHF------------SRVDVAKDDT-------SGSVSIKKIARY-I-KDGNLTTR-FRGGHQIKKFKLVG 
Rep_pFIS3754-01_Fischerella   AKLVDC---------LSGLNLNL------------TRIDLSIDDY-------QKRLNVLDILN--W-FNEGNFSG-FTTKLPIPSGK--- 
Rep_pJTPS1_Ralstoniasol       QAVYAM---------VQDLDARI------------TRCDLAMDFC-------QGEVSIAQMEELYYAGDFNAGGR-IPTYRKIESGVAG- 
Rep_pNL932024_Llactamica      RNLVRD---------VWAEQHAV------------TRIDIAED------------FCEAGLFDEFSARLVSIAKK-NHRLKTSTVGDWLS 
Rep_pFR18_Lmesenteroides      ---------------YSIDHTHL------------TRVDLACD--------------IYNVDLSLY-DFGLFNVT-RDIYRTLSG----- 
Rep_pIH01_Lcitreum            -EQLERVM-------SSVDHAHL------------TRVDLACDI-----------YNIPLTKYD------------FGLFNLTREIRR-- 
Rep_p9785S_Ljohnsonii         AKALRWLL-------DQLQDKRF------------SRLDIA--------------FDMIDLDVKGY-QPYIFGSS-RTIYL--------- 
Rep_pMCCL4_Mcaseolyticus      IR--ENLS-------SVLENVGF------------TRLDLA--------------FDFEEDLSDFF-VMSDNALK-KTVFYGLDG----- 
Rep_pQY003_Emundtii           KNFIH----------DLFLEPHF------------SRADIACDI-----------IDVPDEFITQY-RVVDPVSF-KPIYGRSGK----- 
Rep_p5_Efaecium               KNFIH----------DLFIEPHF------------SRADIACDI-----------IDVPDEFITQY-RVVDPVSF-KPIYGRNGK----- 
Rep_p41-4_Efaecium            KNFIH----------DLFIEPHF------------SRADIACDI-----------IDVPDEFITQY-RVVDPVSF-KPIYGRSGK----- 
Rep_pJS42_Efaecium            KNFIH----------DLFLEPHF------------SRADIACDI-----------VDIPDDFVSQY-RVVDPVSF-KPIYGRSGK----- 
Rep_pRS1_Ooeni                --ILSKI--------ATLKNKKF------------SRCDVAFDI-----------------------FNSPIIEK-YRIFRPGTSSKYFL 
Rep_pF03-3_Lpentosus          ---------------NNMEQAHF------------TRLDIAFDV-----------FN-DDLAMK-Y-RVYRFNTR-EDVIETIKG----- 
Rep_pQJ012_Weissellaconf      ---------------DLLADVHF------------TRLDLAFDV-----------F-NNELGMK-Y-RIYRPNVS-QREYGVYTA----- 

                                                                  Motif I


                                      370       380       390       400       410       420       430       440       450        
                              ....|....|....|....|....|....|....|....|....|....|....|....|....|....|....|....|....|....|
Rel_ICESt3_Sthermophilus      NDN-----MEANHGLSLYFGSRQSQLYFNFYEKRYEIARMENI--SLEESLEIFGIWNRYELRFS--DQKAQGVVEEYI----------- 
Rel_ICE515_Sagalactiae        SDMEEI--EQNRQGISLYFGSRQSEMYFNFYEKRYEIAKQEGI--TVEEALEIFELWNRYEIRLS--QSKANAAVDEFI----------- 
Rel_Tn916_Efaecalis           KE------EKECMGNTLYIGSLQSEVYFCIYEKDYEQYKKNDI--PIEDAEV----KNRFEIRLK--NERAYYAVRDLL----------- 
Rel_ICEBs1_Bsubtilis          --------DGITGGTTIYFGSKKSEAYLCFYEKNYEQAEKYNI--PLEELGD----WNRYELRLK--NERAQVAIDALL----------- 
Rel_Tn6009_Kpneumoniae        KE------EKECMGNTLYIGSLQSEVYFCIYEKDYEQYKKNDI--PIEDAEV----KNRFEIRLK--NERAYYAVRDLL----------- 
Rel_Tn6202_Efaecalis          G-------QRSNKGLSLYFGSRQSPFYLNFYQKDYELAKKEEI--SVEMARQKYGIKNRYEIRLA--DEKAYLFVEYLL----------- 
Rel_ICESmuUA159_Smutans       G-------RFVNQGLTLNFGSRQSALMIRFYQKDYEQALLKDV--SVDYIHEVYNLKNRYELELH--DTKAFDVLKEWY----------- 
Rel_ICE6013_Saureus           --------DGRSKGATFYIGSKQSNLYCRFYEKNYEQAFKRHC--DVEDIGL----WNRYEIQMR--KAYAVNCAKVLS----------- 
Rel_Tn6098_Llactis            G-------FLKSLGISLYFGSIQGFIRFNFYEKDLEQAFRRNI--PVEDVQEIFGFKNRYEIRLK--DDKAFQVIEDFVG--------WE 
Rel_Nisin-sucrose transposon  E-------GMINDGLTVYFGSKQTHLFFRFYEKDYERASQEMT--SVEAIREMYGLRNRYEISMR--KEISTDFIKRYI----------- 
Rel_Tn5386                    HNEA----DKGGMGHTLYIGSFSSEVYFCCYEKNYEQYAKLGI--PIEEVPI----KNRFEIRLK--NERAYYAVRELL----------- 
Rel_ICECp1                    HNEQ----DKAGMGHTLYIGSLKSEVYFCCYEKNYEQYAKLGV--PIEEAPI----KNRFEIRLK--DERAYYAVRELL----------- 
Rel_EfaC2                     K-------KMVNKGLSLYFGSRQSPLYFNFYQKDYELARKESM--SVEEAREKHEIKNRYEIRLS--DEKAFLFVEYFL----------- 
Rel_Tn916_Blongum             HRED----DREYMGRTLYLGSLKSDVYFCIYEKDYEQYVKLGT--PLEEADI----INRFEIRLR--NERAYYAVRDLL----------- 
Rel_Tn916_Ctrachomatis        KE------EKECMGNTLYIGSLQSEVYFCIYEKDYEQYKKNDI--PIEDAEV----KNRFEIRLK--NERAYYAVRDLL----------- 
Rel_Tn916_Mabscessus          AD------EKDGMGNTLYIGSLKSEVYFCLYEKDYEQYIKLGI--PLDKTET----KNRFEIRLK--NDRAYHAIQDLL----------- 
Rel_Tn916_Salboniger          RD------EKPDMGNTLYIGSLKSEVYFCIYEKDYEQYVKNDI--ALEDAEI----KNRFEIRLK--NDRATQAMKDLL----------- 
Rel_ICE_SanC238_tRNAleu       N-------RFVNQGLTLNFGSRQSSLMIRFYQKDYEQALLKDV--SVDYIHEAYNYKNRYEIELH--DTKAFDILKEWY----------- 
Rel_ICE_Sga43143_rpsI         N-------RFVNQGLTLNFGSRQSSLMIRFYQKDYEQALLKDV--SVDYIHEAYNYKNRYEIELH--DTKAFDILKEWY----------- 
Rel_ICE_SintB196_tRNAleu      N-------RFVNQGLTLNFGSRQSSLMIRFYQKDYEQALLKDV--SVDYIHEAYNYKNRYEIELH--DSKAFDILKEWY----------- 
Rel_ICE_Sdy12394_lysS         YSE-----NEDRQGISLYFGSRKSNMFFNFYEKRYEFAQKEGI--SVEEALEIFGVWNRYEIRLS--QAKAQKLVEHYV----------- 
Rel_ICE_SparauNCFD2020_rpsI   MEE-----VDGSQGISLYFGSRQSNLYFNFYEKRYELAKSEQF--SVEESLEIFGIWNRYELRFA--QEKAQLAIEEYI----------- 
Rel_ICE_SgaUCN34_ftsK         SNNDY---EEERQGISIYFGSRQSEMYFNFYEKRYELAKQERI--TVNEGLEVFNIWNRYEIRLA--QKKADSIVNEYI----------- 
Rel_ICE_SmiB6_guaA            RD------EKPYMGNTLYIGSLKSEVYFCIYEKDYEQYVKNDV--PLEDAEV----KNRFEIRLK--NDRATQAMKDLL----------- 
Rel_ICE_SdyRE378_rpsI         NED-----MEANHGLSLYFGSRQSQLYFNFYEKRYELARQENI--SLEESLEIFGIWNRYELRFS--DQKAQGAIEEYI----------- 
Rel_ICE_SparasFW213_ebfC      NED-----MEANHGLSIYFGSRQSQLYFNFYEKRYEIARMENI--SLDESLEIFGIWNRYELRFS--DQKAQGIVEEYI----------- 
Rel_ICESpn8140                NED-----MEANHGLSIYFGSRQSQLYFNFYEKRYEIARMENI--SLDESLEIFGIWNRYELRFS--DQKAQGIVEEYI----------- 
Rel_ICE_Sdy2713_tRNAthr       NDMEEI--EQNRQGISLYFGSRQSEMYFNFYEKRYELAKLEGI--SVEESLEIFEIWNRYEIRLS--QSKANAVVDEFI----------- 
Rel_dICE_Sag2603_tRNAlys      SDMEEI--EQNRQGISLYFGSRQSEMYFNFYEKRYEIAKQEGI--TVEEALEIFELWNRYEIRLS--QSKANAAVDEFI----------- 
Rel_ICE_SgaUCN34_Tn916        KE------EKECMGNTLYIGSLQSEVYFCIYEKDYEQYKKNDI--PIEDAEV----KNRFEIRLK--NERAYYAVRDLL----------- 
Rel_ICE_Sga2069_rpmG          TQEKY---EEERQGISLYFGSRQSEMYFNFYEKRYELAKEERI--SVEEALEIFDVWNRYEIRLA--HKKADSVVDEYI----------- 
RepSTK1-4CIJ                  --------GGETIGHTLYFGAPSSDIQVRFYEKNVQMGMDIDV-------------WNRTEIQLR--DDRAHVVAQIIA----------- 
RepC_pT181                    ------------KPETKYFGVRDSNRFIRIYNKKQERKDNADA--EVMSEH-----LWRVEIELK--RDMVDYWNDCFS----------- 
RepD_pC221                    ------------KPETKYFGVRDSDRFIRIYNKKQERKDNADV--EVMSEH-----LWRVEIELK--RDMVDYWNDCFD----------- 
RepE_pS194                    ------------KPETKYFGVRDSDRFIRIYNKKQERKDNADI--EVMSEH-----LWRVEIELK--RDMVDYWNDCFN----------- 
RepI_pUB112                   ------------KPETKYFGVRDSDRFIRIYNKKQERKDNADV--EVMSEH-----LWRVEIELK--RSMVDYWNDCFN----------- 
RepJ_pC223                    ------------KAETKYFGSRDSNRFIRIYNKKKERKENADV--DVSAEH-----LWRVEIELK--RDMVDYWNNCFN----------- 
RepN_pCW7                     ------------KPETKYFGVRDSDRFIRIYNKKQERKDNADI--KIMSEH-----LWRVEIELK--RDMVDYWNDCFN----------- 
Rep_pRS2_Ooeni                -------------LQTAYFGARSSEKQIRLYNKRIERIKKGRI----EDLKDPEQCYWRLELQLR--RGRADDFQKQVD----------- 
Rep_pK214_Llactis             ------------KPETKYFGVRDSNRFIRIYNKKQERKDNADV--EVMSEH-----LWRVEIELK--RDMVDYWNDCFN----------- 
Rep_pSP197_Staphpasteuri      I-------GNSGIIQTIYVNSRKGDSFMRIYNKKEESIINNNI--DMQRALECSN-WTRFELELK--GTYAHNATKALID--------CE 
Rep_pLA2_10_Plarvae           --------SGESEGHTLYFGHGSSSIQIRIYEKHHERKNKGYE--LYEDLST----WNRVEIQAR--DTRALIMAKYVA----------- 
Rep_pBt1-3_Bthuringiensis     --------QGRLQGSTIYFGSASSKLRWVVYDKLEEQKKKNKDSDSIQGLEF----WTRHELRLK--KERADRAVKQLV----------- 
Rep_pBMB2062_Bthuringiensis   ---------------TVYYGNRNKDGYLKIYDKKKELYNHFKR--MIEEEN-----LTRIEYSWRDSDGVVVDEIRKSP----------- 
Rep_pMC5_Exiguobacterium      -------------LETYYAGGSRSKMRLVMYDKKAERASKQGI--DPEELGEKD--WWRIELRFK--ENEIDRLFSE------------- 
Rep_pUMNLJ21_2_Ljohnsonii     --------TNAFLGRTMYFGSQASDLFCRVYDKTLERKINS----DQKDIPEK---WTRLEMVYR--KDRAKKLVDHMI----------- 
Rep_pBC9801_Bcytotoxicus      VDGEEDKLQYVSDGETWYLGSRTGT-QFRFYDKKAQMNASDLL--H----------WVRCELQLV--DDAATNFVKKAI----------V 
Rep_pMC4_Exiguobacterium      --------TGKGSVQTHYAGGKRSKMNIVMYDKRAEQAYSQGI--DPEELGEKE--WWRIEVRLNSSDEVTRFMNDDTYN--------PF 
Rep_pRKC30SC2_Lamylovorus     --------DNHFLGRTMYFGSQVSEIFCRIYDKTLERKAKSDE--TEIPKN-----WTRLEMVYR--KERATKLVDFLV----------- 
Rep_pJS-B_Nmeningitidis       --------NPTEKGKTLTIGSKHSSVFCRIYDKAKEQGDTSGV------------FWCRFEQQYM--GRNCYLSLDILLS--------PG 
Rep_pAH187_12_Bcereus         VDGEEDKLQYVPDGETWYLGSRSGT-QFRFYDKKAQMNAEDLL--H----------WTRCELQLV--DDAATNFVKNAI----------V 
Rep_pFIS3754-01_Fischerella   --------GNKYTGLTLNCGSRESDKFVRIYD-TFGKHKTKAT-~~~~~~~~~~~~~-RFEGEF-----KDYKAKQIQ--QVLMDFALQT 
Rep_pJTPS1_Ralstoniasol       --------SKGCRGTTFEIGRRANGKMLRAYEKGRQLGNQDSE-------------WVRLEIEFG--AKDRVIPHEILI----------- 
Rep_pNL932024_Llactamica      --------DDAKFGRTLYLGSTKSPIRIRLYEKSKKIANELFYKFDYDHPVGFPIDGVRLELQVRPQKQQRFL---------AAKED--- 
Rep_pFR18_Lmesenteroides      ------------KLETRYWGRRKSERQIRLYDKANERKKHGKKDEIPDWAEE----WWRLEFQFR--QGKVDSWQEEIIEKMSSFHVLAV 
Rep_pIH01_Lcitreum            --------TKSGDLETRYWGAGRSERLIRLYDKKRERTAHKKE----DDIPEWAEDWWRLEFQFR--QGKVEKWEEEILDKMGSFQVLAL 
Rep_p9785S_Ljohnsonii         --------TKSWHVGSIYAGARGSKLQIRFYDKKLERKANHE---ELDCNS-----YWRLEMQLR--GSKTITWYDDCK----------- 
Rep_pMCCL4_Mcaseolyticus      ------------KAETKYFGVRESDRFIRIYNKKQEQKDNADV--EIELEN-----YWRFEIELK--RKRVDEWNSNCF----------- 
Rep_pQY003_Emundtii           -------------LETAYWGSRASERQIRMYNKKLEQETKRKI--VPPEIKT----WWRLELQLR--RGKATDWYAMVH----------- 
Rep_p5_Efaecium               -------------LETAYWGSRSSERQIRMYNKKLEQEKKRKI--VPKEIVS----WWRLELQLR--RGKATDWHAMVY----------- 
Rep_p41-4_Efaecium            -------------LETAYWGSRASERQIRMYNKKLEQERKRKI--VPPEITT----WWRLELQLR--RGKATDWHEMVH----------- 
Rep_pJS42_Efaecium            -------------LETAYWGSRASERQIRMYNKKLEQERKRKI--VPPEITT----WWRLELQLR--RGKATDWHEMVH----------- 
Rep_pRS1_Ooeni                --------SAAGKPETIYYGAQKSEAQIRQYNKLVEQTKKRKT--IPDNIKN----WWRLELQLR--GRKISDYPQQVK----------- 
Rep_pF03-3_Lpentosus          --------R-NKSVETMYWGARKSDQQIRLYNKLVEQKNKQKP--IPAGVES----WARLELQLR--GKKPAEWLNSAT----------- 
Rep_pQJ012_Weissellaconf      --------QWTKAVETIYYGSNSSDQQIRQYNKLVEQTKKNMP--LPDGVEH----WMRLELQLR--GRKPAEWVDCAK----------- 

                                            Motif II      Motif III                   Motif IV
