# Supplementary material for: Characterization of a relaxase belonging to the MOBT family, a widespread family in Firmicutes mediating the transfer of ICEs
Source: Mob DNA. 2019 May 3;10:18. doi: 10.1186/s13100-019-0160-9 (PMC6499999; doi:10.1186/s13100-019-0160-9)
Supplement: Supplementary file 3 — Figure S3. Calibration curve of the gel filtration column. A Sephadex S200 HiLoad 16/60 column (GE Healthcare) was used as a final step of purification of RelSt3 protein. This column was calibrated and allowed us to estimate the apparent molecular weight of RelSt3. See complete legend in Additional file 4. (PPTX 40 kb) [file 13100_2019_160_MOESM3_ESM.pptx]

## Slide 1
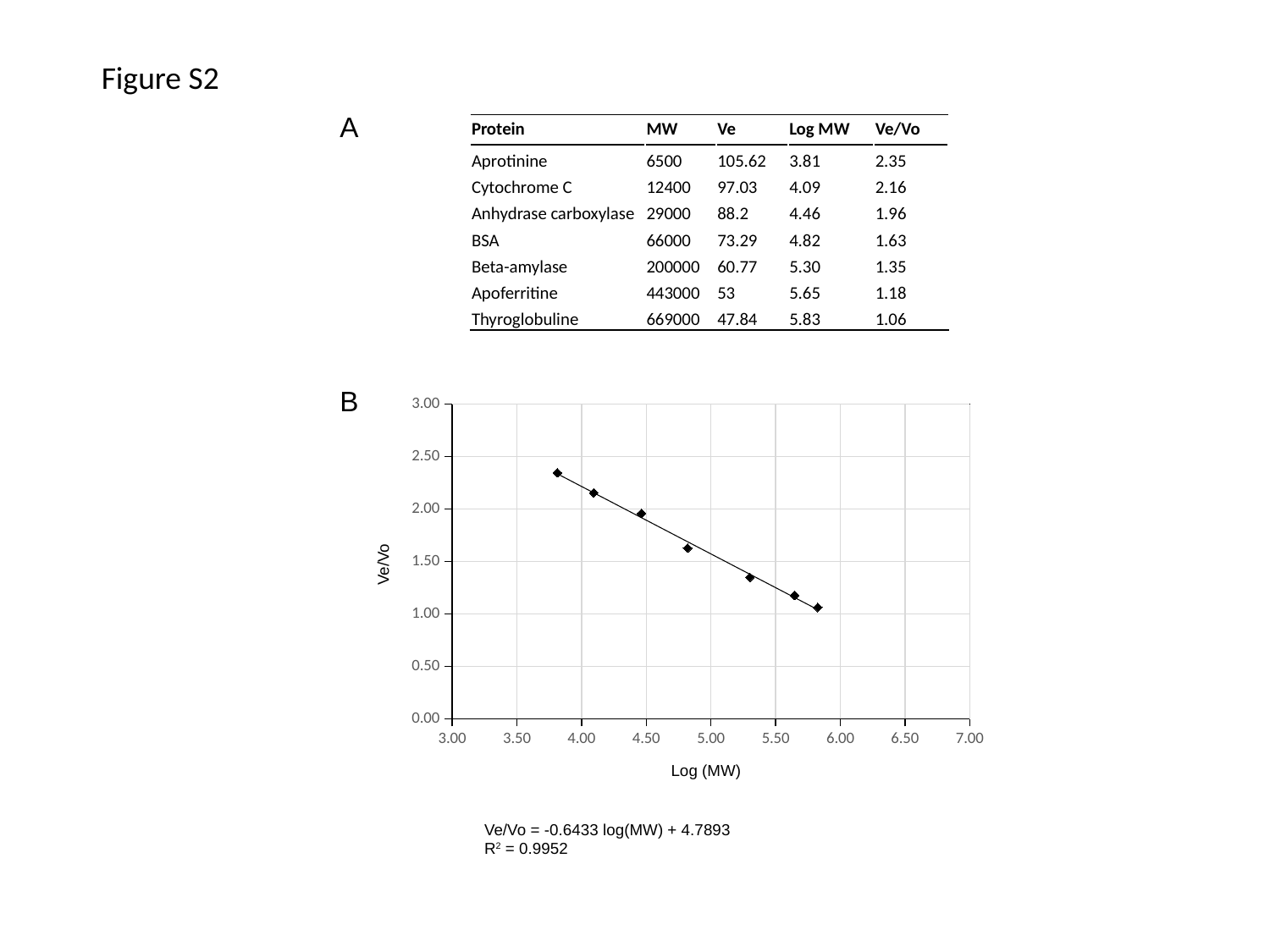

Figure S2
A
| Protein | MW | Ve | Log MW | Ve/Vo |
| --- | --- | --- | --- | --- |
| Aprotinine | 6500 | 105.62 | 3.81 | 2.35 |
| Cytochrome C | 12400 | 97.03 | 4.09 | 2.16 |
| Anhydrase carboxylase | 29000 | 88.2 | 4.46 | 1.96 |
| BSA | 66000 | 73.29 | 4.82 | 1.63 |
| Beta-amylase | 200000 | 60.77 | 5.30 | 1.35 |
| Apoferritine | 443000 | 53 | 5.65 | 1.18 |
| Thyroglobuline | 669000 | 47.84 | 5.83 | 1.06 |
### Chart
| Category | Ve/Vo |
|---|---|B
Ve/Vo
Log (MW)
Ve/Vo = -0.6433 log(MW) + 4.7893
R2 = 0.9952
