# Supplementary material for: Characterization of a relaxase belonging to the MOBT family, a widespread family in Firmicutes mediating the transfer of ICEs
Source: Mob DNA. 2019 May 3;10:18. doi: 10.1186/s13100-019-0160-9 (PMC6499999; doi:10.1186/s13100-019-0160-9)
Supplement: Supplementary file 4 — Supplementary material text. This text includes supplementary Materials and Methods, complete Additional file legends, and tables for bacterial strains, plasmids and oligonucleotides used in this study. (DOC 201 kb) [file 13100_2019_160_MOESM4_ESM.doc]

# Supplementary Material text

Characterization of a relaxase belonging to the MOB_T_ family, a widespread family in Firmicutes mediating the transfer of ICEs

Nicolas Soler, Emilie Robert, Isaure Chauvot de Beauchêne, Philippe Monteiro, Virginie Libante, Bernard Maigret, Johan Staub, David W. Ritchie, Gérard Guédon, Sophie Payot, Marie-Dominique Devignes, and Nathalie Leblond-Bourget

# Supplementary Figure Legend

**Additional file 1: Figure S1. Alignment of MOB_T_ relaxase representatives with *Rep_trans* RCR initiator representatives.** RelSt3: relaxase encoded by ICE*St3* from *S. thermophilus* [1], Rel_ICE_515: relaxase encoded by ICE*_515_tRNA^Lys^* from *S. agalactiae* [2], Orf20_Tn*916*: relaxase encoded by the ICE Tn*916* [3], NicK_ICE*Bs1*: relaxase encoded by ICE*Bs1* [4]. RepSTK1 and RepDE are the sequences of the two *Rep_trans* RCR initiators whose structures have been solved recently (PDB ID: 4CIJ and 4CWC, respectively) [5]. RepC and RepD correspond to the *Rep_trans* proteins from the staphylococcal plasmids pT181 and pC221, respectively [6]. The 5 conserved motifs outlined by Carr *et al* (2016) (Motifs N’, I, II, III, and IV) and the motif N’’ identified in this work for MOB_T_ proteins are boxed. The N’ motif, located in the poorly conserved N-terminal part of the proteins, harbours a conserved aspartic acid that is one of the three acidic residues (labelled with *) that were shown to coordinate a cationic cofactor in *Rep_trans* proteins. The two other acidic residues are located in motif I and motif IV, and are also well conserved in the alignments between MOB_T_ and *Rep-trans* proteins [5]. The conserved catalytic tyrosine of motif III (labelled with #, Y188 in RepD sequence, Y252 in RelSt3 sequence) has been shown to be responsible for the nicking-closing activity of the RepD protein of the pC221 staphylococcal plasmid [7]. The role of motifs N’’ and II is currently unknown. This sequence alignment was made using the Muscle program [8].

**Additional file 2: Figure S2. Sequence alignment of MOB_T_ relaxases and *Rep_trans* RCR initiators.**

A larger alignment with more MOB_T_ and *Rep_trans* initiator sequences was constructed. The same conserved motifs identified in Figure S1 are boxed (Motives N’, N’’, I, II, III, and IV). All sequences included in this alignment are detailed in Table S4.

MOB_T_ proteins: sequences used in Figure S1 with the addition of Rel_Tn*6009* from *Klebsiella pneumoniae* [9], Rel_Tn6202 encoded by *Enterococcus faecalis* (Tn*6202* encoding vancomycin resistance) [10], Rel_ICE_SmuUA159 encoded by ICE_*SmuUA159_tRNAleu*, Rel_Tn6013 from *Staphylococcus aureus* [11], Rel_Tn6098 encoded by *Lactococcus lactis* (Tn*6098* encoding alpha-galactoside degradation) [12], Rel_ICECp1 from *Clostridium perfringens* (ICE*Cp1* encoding bacitracin resistance) [13], Rel_Nisin-sucrose from *Lactococcus lactis* (this conjugative transposon encoding the nisin bacteriocin biosynthesis and also sucrose degradation) [14], Rel_EfaC2 from *Enterococcus faecalis* strain V583 [15], 4 other Tn*916* relaxases from strains of different phyla: Blongum: *Bifidobacterium longum*, Mabscessus: *Mycobacterium abscessus*, Salboniger: *Streptomyces alboniger*, Ctrachomat: *Chlamydia trachomatis*, and 14 sequences of streptococcal ICEs representative of each clustering group identified previously [16].

*Rep_trans* proteins: sequences used in Figure S1 with the addition of *Rep_trans* proteins from 5 other members of the pT181 family of staphylococcal plasmids: RepE (pS194), RepI (pUB112), RepJ (pC223), RepN (pCW7) and the Rep from pKH6 plasmid [6]. 27 other *Rep_trans* proteins from *bona fide* RCR plasmids were recovered by BLAST using RepSTK1 or RepC_pT181 as query, as described in the Supplementary Materials and Methods. The alignment was not shown entirely as the C-terminal parts of the proteins did not align properly. This sequence alignment was made using the Muscle program [8].

**Additional file 3: Figure S3. Calibration curve of the gel filtration column.**

A Sephadex S200 HiLoad 16/60 column (GE Healthcare) was used as a final step of purification of RelSt3 protein. This column was calibrated to estimate the apparent molecular weight of RelSt3. Panel A indicates the different template proteins used to calibrate the column and their corresponding data. Panel B shows the resulting calibration curve plotted as Ve/Vo = f(logMW), where Ve is the elution volume, Vo is the dead volume, and MW, the molecular weight.

#### Supplementary Materials and Methods

##### **Streptococcus thermophilus growth conditions**

*Streptococcus thermophilus* was cultured on M17-lactose 0.5% (referred as LM17) [17] at 30°C or 42°C depending on the selection of the thermosensitive replication vector pG^+^host9. For natural transformation experiments, LM17 and reconstituted chemically defined medium (CDM) supplemented with 0.5% of lactose (LCDM) [18] were used. Erythromycin was added at 150 µg/mL for *E. coli* and 5 µg/mL for *S. thermophilus*. Chloramphenicol resistance selection for *S. thermophilus* was done at 4 µg/mL.

***Construction of ICE*St3 *deleted of* orfJ**

Our strategy was to delete the *orfJ* CDS and the 15 bp of the *orfJ/I* untranslated region while keeping the predicted *oriT* region and other conjugation genes intact.

PCR amplifications of (i) the upstream region of *orfJ* CDS (ΔRel_1/ΔRelATG_2 primers, adding a 5’ *Not*I restriction site to the 1339 bp fragment) and (ii) its downstream region (ΔRelATGorfI_3/ΔRel_4 primers, adding a 3’ *Apa*I restriction site to the 1007 bp fragment) were performed using total DNA isolated from *S. thermophilus* CNRZ385, the original strain carrying ICE*St3* [19]. PCRs were carried out according to the instructions of the Phusion High-Fidelity DNA Polymerase kit (Fermentas). To produce overlapping regions between adjacent fragments, the 20-nucleotides reverse complement sequence of the ΔRelATG_2 primer was added at the 5’ end of the ΔRelATGorfI_3 primer. The PCR fragment deleted for the *orfJ* CDS, resulting from overlapping PCR between fragment (i) and (ii), was generated as described in Lecomte *et al*, 2014 [20]. After gel extraction purification, the overlapping PCR fragment and pG^+^host9 were digested with *Not*I and *Apa*I, with SAP (Shrimp Alkaline Phosphatase) exposure for the plasmid, as recommended by the provider (Fermentas).

pG^+^host9 is a thermosensitive derivative of pWV01 carrying an *Erm*^R^ gene [21]. The digested overlap fragment was cloned into pG^+^host9 and the resulting ligation mixture was used to transform *E. coli* EC101 cells. Erythromycin-resistant transformants were tested by PCR using pGhost9for and HindP primers to detect the 2761 bp fragment expected for the *orfJ* flanking regions. The resulting plasmid (pG^+^host9*ΔorfJ*) was introduced in *S. thermophilus* LMG18311 ICE*St3cat* [1] by natural transformation (adapted from Gardan *et al*, 2009 [22]) and the transformed cells were grown 24 h at 42°C. This temperature is restrictive for pG^+^host9*ΔorfJ* replication and allowed the selection of integrants, *i.e.* clones with a chromosomal copy of the plasmid resulting from a single crossing-over between the conjugation module regions of ICE*St3* carried by the plasmid and the homologous chromosomal regions of LMG18311 ICE*St3cat* upstream or downstream of *orfJ* CDS. As adapted from Biswas *et al*, 1993 [23], integrant clones were grown overnight at 30°C in the presence of erythromycin to favor the chromosomal excision of the plasmid due to a second crossing over between chromosomal duplicated sequences. These clones were then grown at 42°C without plasmid selection for 20 generations to lower the plasmid copy number and promote its loss. The cultures were plated on chloramphenicol resistance selective medium. Colonies were replicated on erythromycin-containing plates. Clones lacking pG^+^host9*ΔorfJ* with or without deletion of the *orfJ* CDS were *Cm*^R^ and *Erm*^S^. Depending on the localization of the two homologous recombination events, these clones could either or not carry the *orfJ* CDS. PCR screening was performed on these clones using primers flanking *orfJ* to determine their genotypes (K/J.For and J/I.Rev primers). A 328 bp PCR product is expected for the Δ*orfJ* mutant and a 1,561 bp amplification product for the WT strain. LMG18311 Δ*orfJ* mutant clones were further verified by sequencing (Cogenics, Beckman Coulter genomics).

***Over-expression and purification of RelSt3 WT and mutant proteins***

The pSKB3 constructs harbouring RelSt3 coding sequences (WT or variants) were used to transform *E. coli* BL21(DE3) cells [24]. Overexpression of the corresponding proteins was done in LB media supplemented with kanamycin (50 µg/mL). Cells were initially grown at 37°C until OD_600_ = 0.5, and then shifted for 5h at 18°C for overexpression induction with a final concentration of 0.5 mM isopropyl-β-D-thiogalactoside. Cells were then collected and stored as a cell pellet at -80°C.

Before purification of RelSt3 proteins, his-tagged TEV protease was overproduced and purified from *E. coli* BL21(DE3) as recommended by van den Berg *et al*, 2006 [25]. Cells overexpressing RelSt3 proteins were suspended in lysis buffer (15 mL/g of cell pellet) containing 20 mM Tris-HCl pH 7.5, 500 mM NaCl, 10 mM imidazole, 0.04 mg/mL lysozyme, 0.1% Triton X100, and 2 mM β–mercapto-ethanol. After 30 min of lysis at 4°C with stirring, cells were sonicated on ice. Clarification of the samples was performed by centrifugation (10,000 g, 1.5 h, 4°C) to remove insoluble materials. The supernatant was then loaded on a 1 mL His-Trap FF column using an ÄKTAprime plus (GE Healthcare), with a 1 mL/min flow. The column was washed with 15 column volumes (CV) of buffer A (20 mM Tris-HCl pH 7.5, 500 mM NaCl, 2 mM β-mercapto-ethanol) + 10 mM imidazole, then washed with 15 CV of buffer A + 50 mM imidazole, and the protein was finally eluted using a 24 CV gradient from 50 to 300 mM imidazole in buffer A. Fractions containing RelSt3 proteins were dialysed overnight against buffer A + 10 mM imidazole. To remove the his-tag from proteins, TEV protease was added to the sample at a mass ratio of 1:5 (His-TEV: RelSt3) and the mixture was incubated 20 h at 4°C. Next, the samples were next loaded by gravity onto a 0.8 mL Ni^2+^-sepharose column (GE Healthcare) to eliminate the cleaved his-tag, his-TEV and any other potential contaminants. Flow-through was dialysed overnight against buffer A. The proteins were then concentrated by centrifugation using an Amicon (Millipore) with 10 kDa of MWCO prior to be loaded on a Superdex 200 pg HiLoad 16/600 (GE Healthcare, 1 mL/min). The homogeneity of the samples was checked by SDS-PAGE analysis.

#### Supplementary Tables

Table S1. Bacterial strains used in this work

| **Strains** | **Genotype or description** | **Source or Reference** |
| --- | --- | --- |
| ***E. coli* strains** | | |
| EC101 | *supE hsd-5 thi* (*lac-proAB*) F (*traD6 proAB lacI*^q^ *lacZ* M15) repA, derivative of strain TG1 (56) *repA*, derivative of strain JM101 | Laboratory stock, [26] |
| DH5α | F^-^ Φ80*lac*ZΔM15 Δ(*lac*ZYA-*arg*F) U169 *rec*A1 *end*A1 *hsd*R17(r_k_^-^, m_k_^+^) *pho*A *sup*E44 *thi*-1 *gyr*A96 *rel*A1 λ^-^ | Invitrogen |
| BL21(DE3) | str. B F^–^ *ompT* *gal* *dcm* *lon* *hsdS_B_*(*r_B_*^–^*m_B_*^–^) λ(DE3 [*lacI* *lacUV5*-*T7p07* *ind1* *sam7* *nin5*]) [*malB*^+^]_K-12_(λ^S^) | Laboratory stock, [24] |
| ***S. thermophilus* strains** | | |
| CNRZ385 | Wild-type strain harboring ICE*St3* | INRA-CNRZ strain collection |
| LMG18311 (ICE*St3cat*) | LMG18311 strain carrying ICE*St3* tagged with the *cat* gene inserted in the pseudogene *Ψorf385J*, Cm^r^ | [1] |
| LMG18311 (ICE*St3*Δ*orfJcat*) | LMG18311strain carrying ICE*St3* deleted for *orfJ* and 15 bp of the *orfJ/I* unstranslated region, and tagged with the *cat* gene inserted in the pseudogene *Ψorf385J*, Cm^r^ | This work |
| LMG18311 (pMG36e) | LMG18311 carrying pMG36e, a plasmid conferring erythromycin resistance | [1] |
| LMG18311 (ICESt3cat, pOri1180spec) | LMG18311 ICE*St3cat* strain carrying pOri1180spec conferring spectinomycin resistance | This work |
| LMG18311 (ICESt3cat, pOri1180-*oriT*) | LMG18311 ICE*St3cat* strain carrying pOri1180-*oriT* conferring spectinomycin resistance and harboring the intergenic region between *orfK* and *orfJ* genes | This work |

**Table S2. Plasmids used in this work**

| **Plasmid** | **Description** | **Source or Reference** |
| --- | --- | --- |
| pTH24 | His-tagged-TEV protease coding sequence under control of T7/lac promoter | [25] |
| pSKB3 | Expression vector derived from pET28a including an in frame 6 His-tag and a TEV protease cleavage site in the N-terminal sequence | Gift from Stephen K. Burley |
| pBR322-*oriT* | pBR322 plasmid with the intergenic *orfJ/orfK* region (including *oriT nic* sequence) of ICE*St3* cloned between *Eco*RI and *Hind*III restriction sites | This work |
| pG^+^Host9 | 3.8 kb, pWV01-type thermosensitive replication origin from pVE6002, Erm^r^ | [21] |
| pG^+^host9Δ*orfJ* | pG^+^Host9 carrying the 1339 bp upstream region of *orfJ* CDS and its 1007 bp downstream region, Erm^r^ | This work |
| pMG36e | 3.4 kb, replication origin from pWV01, Ery^r^ | [27] |
| pOri1180 | pOri23 plasmid modified with a multicloning cassette from pSL1180-speclox conferring spectinomycin resistance | [28] and this work |
| pOri1180-*oriT* | pOri1180 plasmid with ICE*St3* *oriT* sequence cloned between *Eco*RI and *Apa*I restriction sites. | This work |

**Table S3. Oligonucleotides used in this work**

All oligonucleotides were purchased from Eurogentec. Restriction sites are underlined, and initiator codons are indicated with bold characters.

| **Name** | **Sequence** |  |
| --- | --- | --- |
| **Oligonucleotides used for deletion of *orfJ* gene in ICE*St3*** | | |
| deltaRel_1 | GGGCGGCCGCAGGCTCTAAACTCCAACTTATG |  |
| deltaRelATG_2 | **CAT**TCATGGGAACACCTCCT |  |
| deltaRelATGorfI_3 | *AGGAGGTGTTCCCATGA****ATG***GAATACAAAGTCGAAATCA  (the nucleotides overlapping with deltaRelATG_2 are represented in italics) |  |
| deltaRel_4 | CGGGGCCCACTTTATTTGAGAGCGTGATAG |  |
| K/J.For | TGTTCATCATGGGCTAGGAC |  |
| J/I.Rev | AAGGTACTTAGTCCACCTGACC |  |
| pGhost9For | CGCCATACCACAGATGTTCCAGATAAA |  |
| HindP | GACGTTGTAAAACGACGGCCAGT |  |
| **Oligonucleotides used for cloning of *orfJ* gene into pSKB3** | | |
| St3-Rel-For | GGGAACATATGACTAAAATAAGTCCCTTTC |  |
| St3-Rel-Rev | CGCAAAGCTTCTAGTGTTCATAGTGTTTGTTGG |  |
| **Oligonucleotides used for cloning of ICE*St3* *oriT* sequence in pOri1180** | | |
| St3-oriTpOri-For | AAAAAGAATTCTGTTCATCATGGGCTAGG |  |
| St3-oriTpOri-Rev | AAAAAGGGCCCTTGATCTGAAAGGGACTTATTT |  |
| **Oligonucleotides used for cloning of ICE*St3* *oriT* sequence in pBR322** | | |
| St3-oriTpBR-For | AAAAAGAATTCTGTTCATCATGGGCTAGGAC |  |
| St3-oriTpBR-Rev | AAAAAAAGCTTTTGATCTGAAAGGGACTTATTT |  |

**Table S4. MOB_T_ and *Rep_trans* sequences used for the alignment in Fig. S1 and Fig. S2**

| **Protein id** | **Name of ICE or plasmid** | **Organism** |
| --- | --- | --- |
| CAE52362 | ICE*St3* | *Streptococcus thermophilus* CNRZ385 |
|  | ICE_*515_tRNA^Lys^* | *Streptococcus agalactiae* 515 |
| AAB60013 | Tn*916* | *Enterococcus faecalis* |
|  | ICE*Bs1* | *Bacillus subtilis* |
| ACF08822 | Tn*6009* | *Klebsiella pneumoniae* strain 41 |
| ACQ89867 | Tn*6202* | *Enterococcus faecalis* N00-410 |
| AAN57979 | ICE_*SmuUA159_tRNA^leu^* | *Streptococcus mutans* UA159 |
| ACI48613 | ICE*6013* | *Staphylococcus aureus* ST239 |
| ADA65815 | Tn*6098* | *Lactococcus lactis* subsp. *lactis* KF147 |
| WP_015426015 | Nisin-sucrose transposon | *Lactococcus lactis* |
| NP_813946 | ICE*EfaC2* | *Enterococcus faecalis* V583 |
| CRG98343 | ICE*Cp1* | *Closridium perfringens* |
| CRH83902 | Tn*916* | *Chlamydia trachomatis* |
| WP_032684677 | Tn*916* | *Bifidobacterium longum* |
| CPW40522 | Tn*916* | *Mycobacterium abscessus* |
| KUJ31040 | Tn*916* | *Streptomyces alboniger* |
| AGU82651 | ICE_*SanC238_tRNA^leu^* | *Streptococcus anginosus* C238 |
| BAK27188 | ICE_*Sga43143_rpsI* | *Streptococcus gallolyticus* subsp. *gallolyticus* ATCC 43143 |
| AGU77050 | ICE_*SintB196_tRNA^leu^* | *Streptococcus intermedius* B196 |
| ADX24159 | ICE_*Sdy12394_lysS* | *Streptococcus dysgalactiae* subsp. *equisimilis* ATCC 12394 |
| EGE53959 | ICE_*SparauNCFD2020_rpsI* | *Streptococcus parauberis* NCFD 2020 |
| CBI14187 | ICE_*SgaUCN34*_Tn*916* | *Streptococcus gallolyticus* UCN34 |
| CBJ22567 | ICE_*SmiB6_guaA* | *Streptococcus mitis* B6 |
| BAM60435 | ICE_*SdyRE378_rpsI* | *Streptococcus dysgalactiae* subsp. *equisimilis* RE378 |
| AFJ26795 | ICE_*SparasFW213_ebfC* | *Streptococcus parasanguinis* FW213 |
| CBW39306 | ICE*Spn8140* | *Streptococcus pneumoniae* |
| CCI63195 | ICE_*Sdy2713_tRNA^thr^* | *Streptococcus dysgalactiae* subsp. *equisimilis* AC-2713 |
| AAN00876 | dICE_*Sag2603_tRNA^lys^* | *Streptococcus agalactiae* 2603V/R |
| CBI14161 | ICE_*SgaUCN34_ftsK* | *Streptococcus gallolyticus* UCN34 |
| CBZ49399 | ICE_*Sga2069_rpmG* | *Streptococcus gallolyticus* subsp. *gallolyticus* ATCC BAA-2069 |
| BAA06270 | pSTK1 | *Geobacillus stearothermophilus* |
| AAW36316 | pT181 | *Staphylococcus aureus* |
| CAA26104 | pC221 | *Staphylococcus aureus* |
| CAA29842 | pS194 | *Staphylococcus aureus* |
| CAA30291 | pUB112 | *Staphylococcus aureus* |
| AAQ55246 | pC223 | *Staphylococcus aureus* |
| AAA26669 | pCW7 | *Staphylococcus aureus* |
| CAC67505 | pRS2 | *Oenococcus oeni* |
| CAA63520 | pK214 | *Lactococcus lactis* subsp. *lactis K214* |
| CAJ13696 | pSP197 | *Staphylococcus pasteuri* |
| AHD07785 | pLAZ_10 | *Paenibacillus larvae* subsp. *larvae* DSM 25430 |
| AFV53176 | pBt1-3 | *Bacillus thuringiensis* serovar aizawai strain 1-3 |
| AHZ54416 | pBMB2062 | *Bacillus thuringiensis kurstaki* str YBT-1520 |
| AHC04797 | pMC5 | *Exiguobacterium* sp. S3-2 |
| ARW74170 | pUMNLJ21_2 | *Lactobacillus johnsonii* strain UMNLJ21 |
| ABS24210 | pBC9801 | *Bacillus cytotoxicus* NVH 391-98 |
| AHC04796 | pMC4 | *Exiguobacterium* sp. S3-2 |
| ADZ08244 | pRKC30SC2 | *Lactobacillus amylovorus* strain 30SC |
| ACJ82593 | pAH187_12 | *Bacillus cereus* AH187 |
| BAU09055 | pFIS3754-01 | *Fischerella* sp. NIES-3754 |
| BAA32217 | pJTPS1 | *Ralstonia solanacearum* |
| ABC94547 | pNL932024 | *Neisseria lactamica* |
| CAB16640 | pFR18 | *Leuconostoc mesenteroides* subsp. *mesenteroides* strain F552 |
| AAV83491 | pIH01 | *Leuconostoc citreum* |
| AAX56321 | p9785S | *Lactobacillus johnsonii* FI9785 |
| BAH18749 | pMCCL4 | *Macrococcus caseolyticus* JCSC5402 |
| BAO08778 | pQY003 | *Enterococcus mundtii* QU 25 |
| AGS77173 | p5 | *Enterococcus faecium* Aus0085 |
| AQT58651 | p41-4 | *Enterococcus faecium* strain 2014-VREF-41 |
| ABY76206 | pJS42 | *Enterococcus faecium* JH95 |
| CAA07049 | pRS1 | *Oenococcus oeni* |
| AIG59283 | pF03-3 | *Lactobacillus pentosus* strain F03 |
| AKV17188 | pQJ012 | *Weissella confusa* |

#### Supplementary References

1. Bellanger X, Roberts AP, Morel C, Choulet F, Pavlovic G, Mullany P, et al. Conjugative transfer of the Integrative Conjugative Elements ICE*St1* and ICE*St3* from *Streptococcus thermophilus*. J Bacteriol. 2009;191: 2764–2775. doi:10.1128/JB.01412-08

2. Chuzeville S, Puymège A, Madec J-Y, Haenni M, Payot S. Characterization of a new CAMP factor carried by an Integrative and Conjugative Element in *Streptococcus agalactiae* and spreading in streptococci. Biswas I, editor. PLoS ONE. 2012;7: e48918. doi:10.1371/journal.pone.0048918

3. Rocco JM, Churchward G. The integrase of the conjugative transposon Tn*916* directs strand- and sequence-specific cleavage of the origin of conjugal transfer, oriT, by the endonuclease Orf20. J Bacteriol. 2006;188: 2207–2213. doi:10.1128/JB.188.6.2207-2213.2006

4. Lee CA, Grossman AD. Identification of the origin of transfer (*oriT*) and DNA relaxase required for conjugation of the Integrative and Conjugative Element ICE*Bs1* of *Bacillus subtilis*. J Bacteriol. 2007;189: 7254–7261. doi:10.1128/JB.00932-07

5. Carr SB, Phillips SEV, Thomas CD. Structures of replication initiation proteins from staphylococcal antibiotic resistance plasmids reveal protein asymmetry and flexibility are necessary for replication. Nucleic Acids Res. 2016;44: 2417–2428. doi:10.1093/nar/gkv1539

6. Novick RP. Staphylococcal plasmids and their replication. Annu Rev Microbiol. 1989;43: 537–565.

7. Thomas CD, Balson DF, Shaw WV. *In vitro* studies of the initation of staphylococcal plasmid replication. J Biol Chem. 1990;265: 5519–5530.

8. Edgar RC. MUSCLE: a multiple sequence alignment method with reduced time and space complexity. BMC Bioinformatics. 2004;5: 113.

9. Soge OO, Beck NK, White TM, No DB, Roberts MC. A novel transposon, Tn6009, composed of a Tn916 element linked with a Staphylococcus aureus mer operon. J Antimicrob Chemother. 2008;62: 674–680. doi:10.1093/jac/dkn255

10. Boyd DA, Mulvey MR. The VanE operon in Enterococcus faecalis N00-410 is found on a putative integrative and conjugative element, Tn6202. J Antimicrob Chemother. 2013;68: 294–299. doi:10.1093/jac/dks394

11. Sansevere EA, Luo X, Park JY, Yoon S, Seo KS, Robinson DA. Transposase-Mediated Excision, Conjugative Transfer, and Diversity of ICE *6013* Elements in Staphylococcus aureus. O’Toole G, editor. J Bacteriol. 2017;199. doi:10.1128/JB.00629-16

12. Machielsen R, Siezen RJ, van Hijum SAFT, van Hylckama Vlieg JET. Molecular Description and Industrial Potential of Tn *6098* Conjugative Transfer Conferring Alpha-Galactoside Metabolism in *Lactococcus lactis*. Appl Environ Microbiol. 2011;77: 555–563. doi:10.1128/AEM.02283-10

13. Han X, Du X-D, Southey L, Bulach DM, Seemann T, Yan X-X, et al. Functional Analysis of a Bacitracin Resistance Determinant Located on ICE *Cp1* , a Novel Tn *916* -Like Element from a Conjugative Plasmid in Clostridium perfringens. Antimicrob Agents Chemother. 2015;59: 6855–6865. doi:10.1128/AAC.01643-15

14. Rauch PJ, De Vos WM. Characterization of the novel nisin-sucrose conjugative transposon Tn5276 and its insertion in Lactococcus lactis. J Bacteriol. 1992;174: 1280–1287. doi:10.1128/jb.174.4.1280-1287.1992

15. Burrus V, Pavlovic G, Decaris B, Guédon G. Conjugative transposons: the tip of the iceberg. Mol Microbiol. 2002;46: 601–610.

16. Ambroset C, Coluzzi C, Guédon G, Devignes M-D, Loux V, Lacroix T, et al. New Insights into the Classification and Integration Specificity of Streptococcus Integrative Conjugative Elements through Extensive Genome Exploration. Front Microbiol. 2016;6. doi:10.3389/fmicb.2015.01483

17. Terzaghi BE, Sandine WE. Improved medium for lactic streptococci and their bacteriophages. Appl Microbiol. 1975;29: 807–813.

18. Letort C, Juillard V. Development of a minimal chemically-defined medium for the exponential growth of *Streptococcus thermophilus*. J Appl Microbiol. 2001;91: 1023–1029.

19. Bellanger X, Morel C, Decaris B, Guédon G. Derepression of excision of integrative and potentially conjugative elements from *Streptococcus thermophilus* by DNA damage response: implication of a cI-related repressor. J Bacteriol. 2007;189: 1478–1481. doi:10.1128/JB.01125-06

20. Lecomte X, Gagnaire V, Briard-Bion V, Jardin J, Lortal S, Dary A, et al. The naturally competent strain *Streptococcus thermophilus* LMD-9 as a new tool to anchor heterologous proteins on the cell surface. Microb Cell Fact. 13: e82. doi:10.1186/1475-2859-13-82

21. Maguin E, Duwat P, Hege T, Ehrlich D, Gruss A. New thermosensitive plasmid for gram-positive bacteria. J Bacteriol. 1992;174: 5633–5638.

22. Gardan R, Besset C, Guillot A, Gitton C, Monnet V. The oligopeptide transport system is essential for the development of natural competence in *Streptococcus thermophilus* strain LMD-9. J Bacteriol. 2009;191: 4647–4655. doi:10.1128/JB.00257-09

23. Biswas I, Gruss A, Ehrlich SD, Maguin E. High-efficiency gene inactivation and replacement system for gram-positive bacteria. J Bacteriol. 1993;175: 3628–3635.

24. Studier FW, Moffatt BA. Use of bacteriophage T7 RNA polymerase to direct selective high-level expression of cloned genes. J Mol Biol. 1986;189: 113–130.

25. van den Berg S, Löfdahl P-Å, Härd T, Berglund H. Improved solubility of TEV protease by directed evolution. J Biotechnol. 2006;121: 291–298. doi:10.1016/j.jbiotec.2005.08.006

26. Leenhouts K. Integration strategies and vectors. Dev Biol Stand. 1995;85: 523–530.

27. van de Guchte M, Van der Vossen JM, Kok J, Venema G. Construction of a lactococcal expression vector: expression of hen egg white lysozyme in *Lactococcus lactis* subsp. *lactis*. Appl Environ Microbiol. 1989;55: 224–228.

28. Que Y-A, Haefliger J-A, Francioli P, Moreillon P. Expression of *Staphylococcus aureus* clumping factor A in *Lactococcus lactis* subsp. *cremoris* using a new shuttle vector. Infect Immun. 2000;68: 3516–3522.
